# Bioproduction and optimization of newly characterized melanin pigment from *Streptomyces djakartensis* NSS-3 with its anticancer, antimicrobial, and radioprotective properties

**DOI:** 10.1186/s12934-023-02276-y

**Published:** 2024-01-17

**Authors:** Nessma A. El-Zawawy, El-Refaie Kenawy, Sara Ahmed, Shimaa El-Sapagh

**Affiliations:** 1 Department of Botany and Microbiology, Faculty of Science, Tanta University, Tanta, Egypt; 2 Chemistry Department, Polymer Research Unit, Faculty of Science, Tanta University, Tanta, Egypt

**Keywords:** Melanin bioproduction, Actinobacteria, Response surface optimization, Radioprotection, Antimicrobial

## Abstract

**Background:**

Melanin is a natural pigment that is considered a promising biomaterial for numerous biotechnological applications across several industries. Melanin has biomedical applications as antimicrobial, anticancer, and antioxidant properties. Additionally, in the pharmaceutical and cosmetic industries, it is used in drug delivery and as a radioprotective agent. Also, melanin has environmental uses in the fields of bioremediation and the food industry. The biosynthesis of melanin pigment is an area of interest for researchers due to its multifunctionality, high compatibility, and biodegradability. Therefore, our present work is the first attempt to characterize and optimize the productivity of melanin pigment from *Streptomyces djakartensis* NSS-3 concerning its radioprotection and biological properties.

**Results:**

Forty isolates of soil actinobacteria were isolated from the Wadi Allaqui Biosphere Reserve, Egypt. Only one isolate, ACT3, produced a dark brown melanin pigment extracellularly. This isolate was identified according to phenotypic properties and molecular phylogenetic analysis as *Streptomyces djakartensis* NSS-3 with accession number OP912881. Plackett–Burman experimental design (PBD) and response surface methodology (RSM) using a Box-Behnken design (BBD) were performed for optimum medium and culturing conditions for maximum pigment production, resulting in a 4.19-fold improvement in melanin production (118.73 mg/10 mL). The extracted melanin pigment was purified and characterized as belonging to nitrogen-free pyomelanin based on ultraviolet–visible spectrophotometry (UV–VIS), Fourier transform infrared (FT-IR), Raman spectroscopy, scanning electron microscopy (SEM), energy dispersive X-ray spectroscopy (EDX), and NMR studies. Purified melanin demonstrated potent scavenging activity with IC_50_ values of 18.03 µg/mL and revealed high potency as sunscreens (in vitro SPF = 18.5). Moreover, it showed a nontoxic effect on a normal cell line (WI38), while it had a concentration-dependent anticancer effect on HCT116, HEPG, and MCF7 cell lines with IC_50_ = 108.9, 43.83, and 81.99 µg/mL, respectively. Also, purified melanin had a detrimental effect on the tested MDR bacterial strains, of which PA-09 and SA-04 were clearly more susceptible to melanin compared with other strains with MICs of 6.25 and 25 µg/mL, respectively.

**Conclusion:**

Our results demonstrated that the newly characterized pyomelanin from *Streptomyces djakartensis* NSS-3 has valuable biological properties due to its potential photoprotective, antioxidant, anticancer, antimicrobial, and lack of cytotoxic activities, which open up new prospects for using this natural melanin pigment in various biotechnological applications and avoiding chemical-based drugs.

**Supplementary Information:**

The online version contains supplementary material available at 10.1186/s12934-023-02276-y.

## Introduction

There is a wide variety of actinomycetes, all of which are gram-positive and filamentous bacteria. They are found in natural and artificial environments and play an important role in the degradation of organic matter in soil [1]. These saprophytic, free-living bacteria are considered a major source in the development of antibiotics [2, 3]. These microorganisms are the most economically and biotechnologically significant because of their ability to produce a wide range of different pigments and bioactive secondary metabolites [4, 5].

The toxic effects of synthetic pigments have made those derived from microorganisms increasingly appealing [6]. This is because microbial pigments are more reliable, readily available, simple to harvest, and productive [7]. Natural pigments produced by microorganisms such as bacteria, fungi, and yeasts have many advantages in different fields when compared to their synthetic counterparts [8]. There are numerous industrial and medical uses for microbial pigments because they are safe, biodegradable, and non-carcinogenic [9]. Different pigments, including bacteriochlorophylls, flavins, melanin, carotenoids, phenazine, astaxanthin, and ß-carotene, are byproducts of microbial metabolism [10].

Natural pigments, including melanin pigments, can be produced by actinomycetes, which are also capable of producing a wide variety of these pigments [11]. Moreover, melanin can be made by a wide variety of bacteria, including *Pseudomonas*, *Aeromonas*, *Azotobacter*, *Mycobacterium*, *Bacillus*, and *Streptomyces* [12]. *Streptomyces* spp. are the most extensively studied actinomycetes species for melanin production. The classification of melanin pigments is based on their chemical compositions, namely eumelanin, pheomelanin, pyomelanin, and allomelanin [13]. *Auricularia auricula*, a marine strain of *Streptomyces* sp., and the soil fungus *Cladosporium cladosporioides* were shown to produce extracellular melanin mostly composed of pheomelanin. Moreover, certain bacteria, fungi, and other species may produce the black-brown pigment known as pyomelanin. *Ralstonia pickettii*, *Pseudomonas aeruginosa*, and *Streptomyces avermitilis* are significant wild species of bacteria that have been linked to pyomelanin synthesis [14].

The use of microorganisms to produce melanin has proven to be a cost-effective and ecologically sound substitute for the chemical synthesis of melanin [15]. Melanin is a complex hydrophilic polymer with an irregular structure and a negative charge. In many organisms, it is synthesized via the oxidative polymerization of phenolic or indolic compounds [16]. Microorganism-derived pigments are preferred over plant-based pigments because their production is less susceptible to environmental factors [17]. Generally, the majority of microbial melanins are produced by converting either tyrosine (by the DOPA-pathway) or malonyl-coenzyme A (via the DHN-pathway), with the help of distinct groups of enzymes. The first process involves the conversion of the melanin precursor, tyrosine, into L-Dopa, which is then transformed into dopaquinone by the action of tyrosinase and laccase enzymes. The second process involves the internal production of the precursor, malonyl-coenzyme A, by the action of polyketide synthases. This is achieved by the step-by-step decarboxylative condensation of five molecules of malonyl-coenzyme A, resulting in the formation of 1,3,6,8-tetrahydroxynaphthalene (THN) [18]. Microorganisms that use the DOPA-pathway are preferred for high-yield melanin synthesis [19].

Melanin's phenol group, or indole structure, gives it a variety of biological actions in addition to its chemical and physical characteristics. Prior studies have mostly examined the ability of melanin to scavenge free radicals, chelate metal ions, and exhibit antioxidant and antibacterial properties. Additionally, these studies have discussed the many applications of melanin in industries such as agriculture and food production [20, 21]. Applied studies have gradually focused on biomedical aspects, such as magnetic resonance imaging (MRI) contrast agents [31, 32], melanin's anti-tumor [22], immunomodulatory [23, 24], antimicrobial [25, 26], radiation protection [27], photothermal properties [28, 29], and other activities that have gradually attracted attention in recent years [30].

The physiochemical and nutritional parameters, in particular, must be optimized for microbial growth and melanin production. It is challenging and time-consuming to implement a "one factor at a time" optimization method [22]. Several different mathematical techniques with multiple independent variables, like the response surface methodology (RSM) and the Plackett–Burman design (PBD) [33] were used to improve optimization, in which interaction effects between the variables can also be predicted for relatively large-scale production of microbial melanins, which could replace current commercial melanin [34]. Therefore, the main objective of this study is to produce, optimize, and characterize melanin pigment from *Streptomyces djakartensis* NSS-3 for the first time, in addition to assessing its radioprotection and biological properties.

## Materials and methods

### Soil sampling and isolation of actinobacterial strains

Soil samples were collected randomly from Wadi-Allaqui Biosphere Reserve, an extremely arid region which is about 180 km south of Aswan on the eastern side of Lake Nasser, Egypt. It lies between 22° 00 23° 00 N latitudes, 31° 01 and 32° 80 E longitudes (Additional file 1: Fig. S1). This region is inhabited by specifically adapted microorganisms that produce different secondary metabolites, enabling them to survive under extreme environmental conditions. Samples were collected aseptically from 10 to 15 cm below the ground surface. Then, directly delivered to the lab, where they were air dried for 24 h at 45 °C at the Faculty of Science, Tanta University, Tanta, Egypt. For bacteriological analysis, one gram of each soil sample was suspended in 9 ml of distilled water, then vortexed, and diluted serially up to 10^−5^ dilution using the standard serial dilution plate method [35]. After that, one mL of each dilution was plated on starch casein agar (SCA) (Hi-Media, India) in triplicate. Filter-sterilized cycloheximide (75 g/mL) and nystatin (25 g/mL) were added to SCA medium to prevent the growth of molds and yeasts [36]. The cultured plates were incubated for 7–14 days at 30 °C. After being cultured, suspected colonies of actinobacteria were purified for further assays.

### Screening for the most potent melanin-producing actinobacterium and its morphological, physiological and molecular characterization

The isolated actinobacteria were screened for the production of melanin by cultivating on peptone yeast extract iron agar (PYIA) (peptic digest 15 g/L, peptone 5 g/L, yeast extract 1 g/L, ferric ammonium citrate 0.5 g/L, dipotassium phosphate 1 g/L, sodium thiosulphate 0.08 g/L, and distilled water 1 L; agar 20 g; pH 7.2). Plates were incubated at 30 °C for 5–7 days. Pigment production was observed every 24 h regularly, as observation of brown to the black coloration around the colony indicates melanin production [12]. The most potent melanin-producing actinobacterium was coded as ACT3 and subjected to further identification and melanin extraction.

The morphological characteristics of the chosen isolate (ACT3) were studied using macroscopic and microscopic techniques. The isolate was initially identified through macroscopic characterization on International Streptomyces Project (ISP) media types ISP-2, ISP-3, ISP-4, and ISP-6 [37]. Aerial and substrate mycelia, color, and production of diffusible pigments were all observed visually. Scanning electron microscopy (SEM; JEOL, JSM-5200 LV, Electron Microscope Unit, Faculty of Medicine, Tanta University, Egypt) was used for microscopic identification. Strain growth characteristics were analyzed by growing the strain at varying temperatures (25, 35, 45, and 55 °C), pH levels (5.0, 7.0, 9.0, and 10.0), and NaCl concentrations (3.0%, 5%, 7%, and 10% w/v). Experiments were carried out according to Bergey's Manual of Bacteriology to identify the biochemical and physiological characteristics of the strain [38].

The selected isolate ACT3 was molecularly characterized by sequencing its 16S rDNA gene, as described by El-Zawawy, et al. [39]. Cultures were sent to the Molecular Biology Research Unit at Assiut University, where DNA was extracted using a patho-gene-spin DNA/RNA extraction kit manufactured by the Korean firm Intron Biotechnology. With the 27 F (forward) primer (5-AGAGTTTGATCMTGGCTCAG-3) and 1492 R (reverse) primer (5-TACGGCTACCTTGTTACGACTT-3), bacterial DNA samples were sent to SolGent Company in Daejeon, South Korea, for PCR and rRNA gene sequencing. The PCR amplification was carried out in a manner similar to that described by Ali, et al. [40]. Sequencing was performed on the isolated amplification products. The obtained sequences were analyzed with the help of BLAST, a local alignment search tool hosted on the NCBI database by the National Center for Biotechnology Information. GenBank (http://www.ncbi.nlm.nih.gov/genbank) now contains the sequences. In order to determine whether or not the obtained sequences were similar to those already in the NCBI database, a BLAST search was performed. MEGA 7.0 was used to generate the 16s rDNA phylogenetic tree via a neighbor-joining method [41].

### Melanin production and quantification

In order to produce a dark melanin pigment, the selected isolate ACT3 (10^8^ colony forming unit CFU/mL) was inoculated into modified peptone yeast extract iron broth (PYI) supplemented with 2 g/L of L-tyrosine and incubated at 30 °C for 7 days with shaking at 160 rpm [42]. After incubation time, selected cells were collected by centrifugation at 3500 rpm for 15 min and melanin pigment production was quantitatively evaluated by measuring optical density (OD_280_) of the filtrate spectrophotometrically at 280 nm [22]. The components of the medium are essential in melanin production and optimization. Therefore, Plackett–Burman design (PBD) and Box-Behnken design (BBD) were used to find out the role that every component played to optimize melanin production.

### Melanin optimization

#### Screening for the main factors affecting melanin production by Plackett–Burman experimental design (PBD)

Optimization of the medium for maximal melanin pigment production using the selected isolate ACT3 through statistical analysis was done using Plackett–Burman design (PBD) as a first optimization step to select the most critical factors that have positive and significant effects on melanin production. The statistical software used was Design-Expert 7.0 (Stat Ease Inc., Minneapolis, U.S.A). Plackett–Burman design is a sort of two level fractional factorial design, which identifies the most critical environmental conditions and media components from a group of candidates by applying the few numbers of tests. PB design used to detect which elements required for elevated melanin production by selected isolate [43]. In this study, a total of 13 process parameters affecting melanin yield and six dummy variables were analyzed in 20 trials to calculate the standard error. These variables including incubation period, pH, temperature, inoculum size, agitation speed, yeast extract, peptone, peptic digest, L-tyrosine, copper sulphate, ferric ammonium citrate, dipotassium phosphate, and sodium thiosulphate; were added at two levels: low (−1) and high (+ 1) as in Additional file 1: Table S1. The experiment was carried out in 20 runs to investigate the effect of the specified variables on the formation of melanin. All trials were done in triplicate, and the average of melanin production was used as the response. The statistical significance of the first-order model was determined using Fisher’s test for analysis of variance (ANOVA) [44]. Plackett–Burman experimental design is based on the first order model:

$$ {\text{Y }} = \, \beta_{0} + \, \sum \beta_{{\text{i}}} {\text{X}}_{{\text{i}}} $$

where, Y is the response or dependent variable (melanin production); it will always be the variable we aim to predict, β0 is the model intercept and βi is the linear coefficient, and Xi is the level of the independent variable which will help us explain melanin production.

### Response surface methodology using Box Behnken design (BBD)

According to the Plackett–Burman design results, response surface methodology (RSM) with Box–Behnken experimental design (BBD) was conducted to gain optimal levels of the most three significant factors positively enhancing the production of melanin. The optimal levels of these three variables for maximum melanin production were determined by generating response surface graphs to visualize the effect of each variable alone and in combination. Each variable at three levels (coded − 1, 0, and + 1 for low, intermediate, and high values, respectively) (Additional file 1: Table S2) was used to fit a polynomial model [45]. The whole design was composed of seventeen experimental trials with five central points to estimate the repeatability of the method. All trials were performed in triplicate, and the average melanin yield was used as a response. The data were analyzed by multiple regression analysis using Design Expert software, and then the polynomial equation to represent melanin yield as a function of the independent variables tested was derived.

$$ {\text{Y }} = \, \beta_{0} - \, \sum \beta_{{\text{i}}} {\text{X}}_{{\text{i}}} + \sum \beta_{{{\text{ij}}}} {\text{X}}_{{\text{i}}} {\text{X}}_{{\text{j}}} + \, \sum \beta_{{{\text{ii}}}} {\text{X}}_{{\text{i}}}^{{2}} $$

where y is the predicted response, β0 is the intercept term, β*i* is the linear coefficient, β*ij* is the quadratic coefficient, β*ii* is the interaction coefficient, and *XiXj* represent the independent variables [46]. Three-dimensional surface plots were used to express the fitted polynomial equation. To maximize the response, the level of each variable was optimized using the design expert numerical optimization method.

#### Experimental validation

To detect the validity of the model and find out the accuracy and stability of the model, one predicted trial, estimated by the BBD numerical optimization, was selected as a check point and tested experimentally to calculate the percent of deviation. For further validation of the results obtained using BBD, a comparative analysis was carried out on melanin production before and after optimization (under the optimal conditions predicted by the model).

### Extraction and purification of melanin

**Fig. 1:**
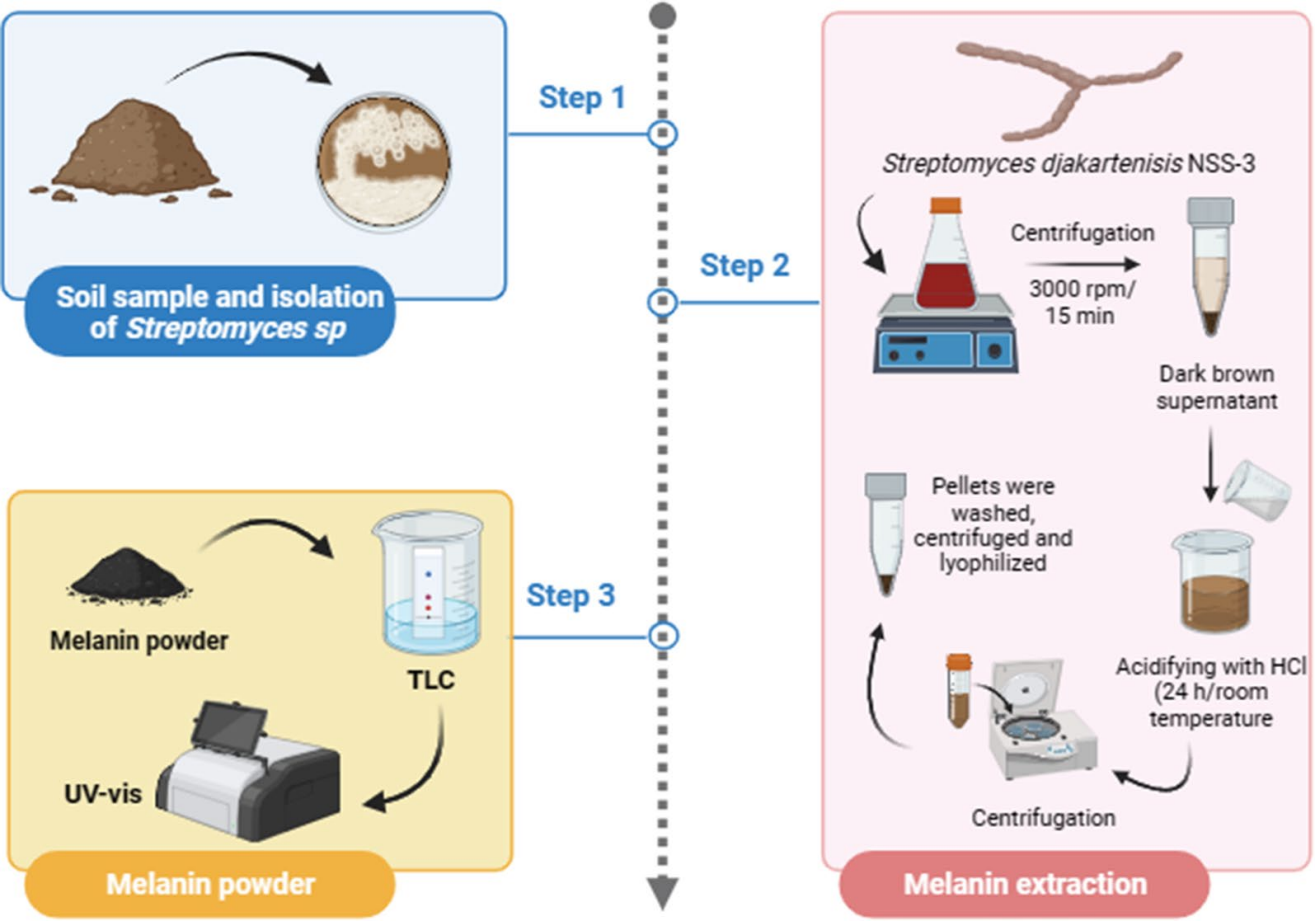
Schematic diagram of melanin extraction from *S. djakartensis* NSS-3

After optimization of melanin production from selected isolate ACT3, melanin was extracted as in Fig. 1 by centrifuging the darkly pigmented medium at 3500 rpm for 15 min (using a refrigerated Eppendorf 5810R) and separating the bacterial cells (pelleted) from the supernatant [22]. The crude pigment granules (melanin) were separated by acidifying the supernatant with 3 mol/L HCl to pH 3, at room temperature for 24 h, and then centrifuging it at 3500 rpm for 15 min. To obtain the pure pigment, melanin pellets were washed with distilled water four times before being centrifuged once more. Lyophilization and long-term storage at 20 °C allowed the purified pigment to be preserved for later physiochemical and spectroscopic studies [47]. Thin-layer chromatography (TLC) was used to confirm the purified melanin [48]. The purified pigment was compared with standard synthetic melanin (Sigma-Aldrich R) using a silica gel chromatography plate (Merck TLC Silica Gel 60 F254) as the stationary phase and the solvent system (Petroleum ether: Ethyl acetate: 95% Ethanol: Ammonia, 4: 4: 6: 1, v/v) as the mobile phase. The purified pigment and standard melanin were spotted on TLC. The developed plates were dried in an oven, the spots were visualized under a UV chamber, and the retention factor (R_f_) value for purified melanin was calculated and compared to the standard one. After scratching the melanin band from TLC, physiochemical and spectroscopic analysis were carried out to confirm the purification of melanin.

### Physiochemical and spectroscopic analysis of melanin

Physiochemical analyses of the purified melanin pigment were carried out using a modified approach mentioned by Muthuraj, et al. [49]. The solubility pattern of the purified melanin pigment was tested by dissolving melanin (5mg/ml) in various inorganic and organic solvents including distilled water, 1N NaOH, 1N HCl, dimethyl sulfoxide as well as methanol, absolute ethanol, chloroform, benzene, acetone, and ethyl acetate. The dissolution was observed after standing for 30 min. For the analysis of bleaching, KMnO_4_ were utilized. Also, in order to determine if the pigment precipitated, 3N HCl and a 1% FeCl_3_ solution were utilized. Moreover, the heat stability of purified melanin was scanned by subjection of melanin to various temperatures at 60, 80, and 100 °C and incubating for 2, 4, and 6 h at the respective temperatures. All of these tests were done compared to standard synthetic melanin.

Ultraviolet (UV) analysis with a Perkin Elmer Lambda 4B UV–vis spectrophotometer was used to verify the melanin purification process [50]. All of the chemical functional groups in purified melanin were identified by performing an FT-IR analysis with an IR spectrophotometer (Perkin-Elmer 1430) [51]. Using a Bruker RFS 27 spectrometer, FT-Raman spectroscopy (SENTERRA II RAMAN microscopy, Bruker) has reported and assigned a wide variety of vibrational modes in purified melanin sample [52]. Melanin's 13 C and 1H NMR spectra were recorded using a JEOL GSX 400 instrument at 30 °C in order to confirm the presence of the functional groups [39].

Energy Dispersive X-ray Spectroscopy (EDX) was used to permit qualitative and quantitative analysis of elemental composition of purified melanin sample [53]. After fixing the melanin samples for 2 h in 2% glutaraldehyde in 0.1 M sodium cacodylate buffer, the samples were washed in the same solution and allowed to dry. Dried sample was metalized with gold (Denton Vacuum Desk V) and then mounted in aluminum sample holders. Scanning electron microscopy (JEOL, JSM-5200 LV) was then used to examine the purified melanin sample.

### Radioprotection measurement

In order to determine the sun protection factor (SPF), the absorbance in the 290–320 nm spectrum was measured in an ethanol solution containing 100 mg of purified melanin pigment from selected isolate ACT3. The SPF value of the purified melanin has been determined using the Mansur equation [54].

$$ {\text{SPF}} = {\text{CF x A }}\left[ \lambda \right]{\text{ x I }}\left[ \lambda \right]{\text{x EE }}\left[ \lambda \right] $$

where CF = 10 is the correction factor, A is the melanin absorbance, λ is the wavelength, I is the solar intensity spectrum, and EE is the erythema effect.

### Antioxidant capacity

The antioxidant activity of purified melanin pigment from selected isolate ACT3 was assessed using DPPH (2, 2-diphenyl-1-picrylhydrazyl) assay according to Singh [55]. In brief, four milligrams of 0.02 mM DPPH was dissolved in 100 ml of methanol and stored at 4 °C to serve as a standard stock solution. Two milliliters of freshly made stock solution were combined with one milliliter of purified melanin pigment at various concentrations (1.25, 2.5, 5, 10, 25, 50, and 100 g/mL). The experiment's negative control was methanol, and the positive control was ascorbic acid (Vitamin C). The absorbance of the mixtures was measured at 517 nm after 30 min of incubation at room temperature in the dark. The free-radical scavenging activity (%) was calculated using the formula:

$$ \left[ {\left( {\text{Absorbance at blank}} \right) \, - \, \left( {\text{Absorbance at test}} \right)/ \, \left( {\text{Absorbance at blank}} \right)} \right]{\text{ x1}}00 $$

The concentration of melanin required to scavenge 50% of the radicals (IC_50_) was determined using a linear regression curve.

#### In vitro cytotoxicity and anticancer activity

Both safety and anticancer activity of the purified melanin of selected isolate ACT3 were measured in vitro on both non-cancerous cells (Human lung fibroblast (WI38) and cancerous cells (Colorectal carcinoma Colon cancer (HCT116), Mammary gland Breast cancer (MCF-7) and Hepatocellular carcinoma (HEPG-2) which obtained from American Collection of Cell Culture (ATCC) via a holding company for biological products and vaccines (VACSERA), Cairo, Egypt. Different cell lines were used to determine the inhibitory effects of purified melanin pigment on cell growth using standard 3-(4, 5 dimethythiazol-2-yl)-2, 5-diphenyl tetrazolium bromide (MTT) assay [56]. This colorimetric assay is based on the reduction of the yellow tetrazolium bromide (MTT) (Sigma Aldrich, USA), to a purple formazan product. The cells were cultured in RPMI-1640 medium (Sigma co., St. Louis, USA) supplemented with 10% fetal bovine serum (GIBCO, UK). Antibiotics added were 100 U/mL penicillin and 100 μg/mL streptomycin at 37 °C in a 5% CO2 incubator. The cells were seeded at a density of 1 × 10^4^ cells/well in a 96-well plate at 37 °C for 48h under 5% CO2. After incubation, the cells were treated with different concentrations (0.05, 0.5, 5, 50 and 500 μg/mL) of purified melanin. Following 24 h of incubation, 0.02 mL of MTT solution (5 mg/mL) was added to each well and incubated for 4 h in a humidified atmosphere of 5% CO2. Purple formazan crystals formed due to MTT reduction by viable cells in each well was dissolved in 100 μL of dimethyl sulfoxide (DMSO) (Sigma Aldrich, USA). Cells that had not been treated with purified melanin pigment were used as a control. The absorbance at 570 nm was measured using a plate reader (EXL 800, USA). Cell viability percentage (CV%) was calculated using the formula:

$$ {\text{CV}}\% \, = \,\left( {{\text{Absorbance of treated samples}}/{\text{Absorbance of untreated sample}}} \right)\, \times \,{1}00 $$

Doxorubicin was used as a standard anticancer drug for comparison.

### Antibacterial activity.

Four multidrug-resistant bacterial strains obtained from our prior studies were used in the current study. Strains of *Pseudomonas aeruginosa* (PA-09), *Escherichia coli* (EC-03), *Klebsiella pneumoniae* (KP-01), and *Staphylococcus aureus* (SA-04) were isolated and identified [51, 57, 58]. The agar-well diffusion method, as described by Ali, et al. [59], was used to estimate the antibacterial activity of purified melanin pigment against the tested strains of bacteria. Fresh overnight cultures of tested strains, approximately 1 × 10^8^ CFU/ml; were used. One hundred microliters of cultural suspension of each tested strain were swabbed unevenly onto individual sterile Mueller–Hinton agar (MHA) (Hi-Media, India) plates by sterile cotton swabs. The suspension of melanin at a concentration of 1 mg/mL was prepared by suspending 10 mg of purified melanin in 10 mL of 1% pure dimethyl sulfoxide (DMSO; Sigma-Aldrich, St. Louis, Missouri, USA) and using it as a stock solution for further studies. Then, five wells of 6 mm diameter were made using a sterilized steel well borer. About 50 μL of melanin solutions with various concentrations (10, 20, 30, 40, and 50 μg/mL) were pipetted into the corresponding wells. The plates were incubated at 30 °C for 24 h, and an inhibition zone appeared around the well, indicating the bioactivity of purified melanin pigment. The diameters of the inhibition zones around the respective wells were measured using a metric ruler, expressed as the mean value (in mm), and compared with the antibiotics streptomycin and tetracycline as positive controls, and DMSO (1%) as a negative control. Triplicates were maintained, and average values were calculated.

### Minimum inhibitory concentration (MIC) and minimum bactericidal concentration (MBC) determination

Following an overnight incubation of selected strains at 37 °C in MHB, turbidity was set to about 10^6^ CFU/mL. The broth microdilution assay was used to determine the MIC and MBC of purified melanin, as described by El-Zawawy, et al. [51]. Purified melanin was prepared by making two-fold serial dilutions in sterile Luria Bertani broth medium, with concentrations ranging from 1.56 g/mL to 200 g/mL. Placed 100 µL of the melanin dilutions in each well of a 96-well microtiter plate, inoculated each well with 100 µL of the test organism in LB at a final concentration of 10^5^ CFU/mL, and incubated at 37 °C with 120 rpm shaking for 24 h. The minimal inhibitory concentration (MIC) of purified melanin was determined as the concentration required for complete inhibition of the growth of pathogenic bacteria after 24 h of incubation at 37 °C. Ten microliters of each set were streaked onto an MHA plate, and the plates were incubated at 37 °C for 24 h to determine the MBC. Since the minimal inhibitory concentration (MIC) of purified melanin defined the 99.5% mortality of selected isolates, we examined the plates to find the MBC that completely inhibited bacterial growth [57].

## Results and discussion

Soil actinomycetes are able to produce a wide variety of products, including enzymes, bioactive secondary metabolites, melanin, and antibiotics [1] that cannot be produced by other types of bacteria because of their unique environment. Actinobacteria are highly desirable due to their potent production capacity of bioactive compounds [60]. Melanin is a promising biomaterial with numerous biotechnological applications in the pharmaceutical, medical, and environmental sectors [22]. Therefore, the first step in the present study was to screen for the most potent melanin producers from soil actinobacteria.

### Actinobacterial isolation and identification of melanin-producing isolates

A total of forty different actinobacteria were selected from soil samples collected from the Wadi Allaqui Biosphere Reserve. Out of forty strains tested in a preliminary screening for melanin production, only isolate ACT3 showed melanin production. The most promising isolate, ACT3, was selected as a potential isolate for the production of melanin, identified on the basis of morphological, cultural, physiological, and biochemical properties. By observing sporulation and the development of vegetative and aerial mycelium, ACT3 was isolated on various ISP media. Different colors were observed in the mycelia of this strain, indicating that it can produce pigments (Additional file 1: Table S3). ACT3 was found to be a gram-positive bacterium with typical growth and sporulation by physiological and biochemical analysis. The strain survived successfully at 25 °C, 5% NaCl, and pH = 7, according to the results. In addition, starch, gelatin, casein, and urea were all found to be degradable by the strain (Additional file 1: Table S4). Further, it was observed that the strain had the ability to degrade the following substrates: starch, gelatin, casein, and urea (Additional file 1: Table S4).

**Fig. 2:**
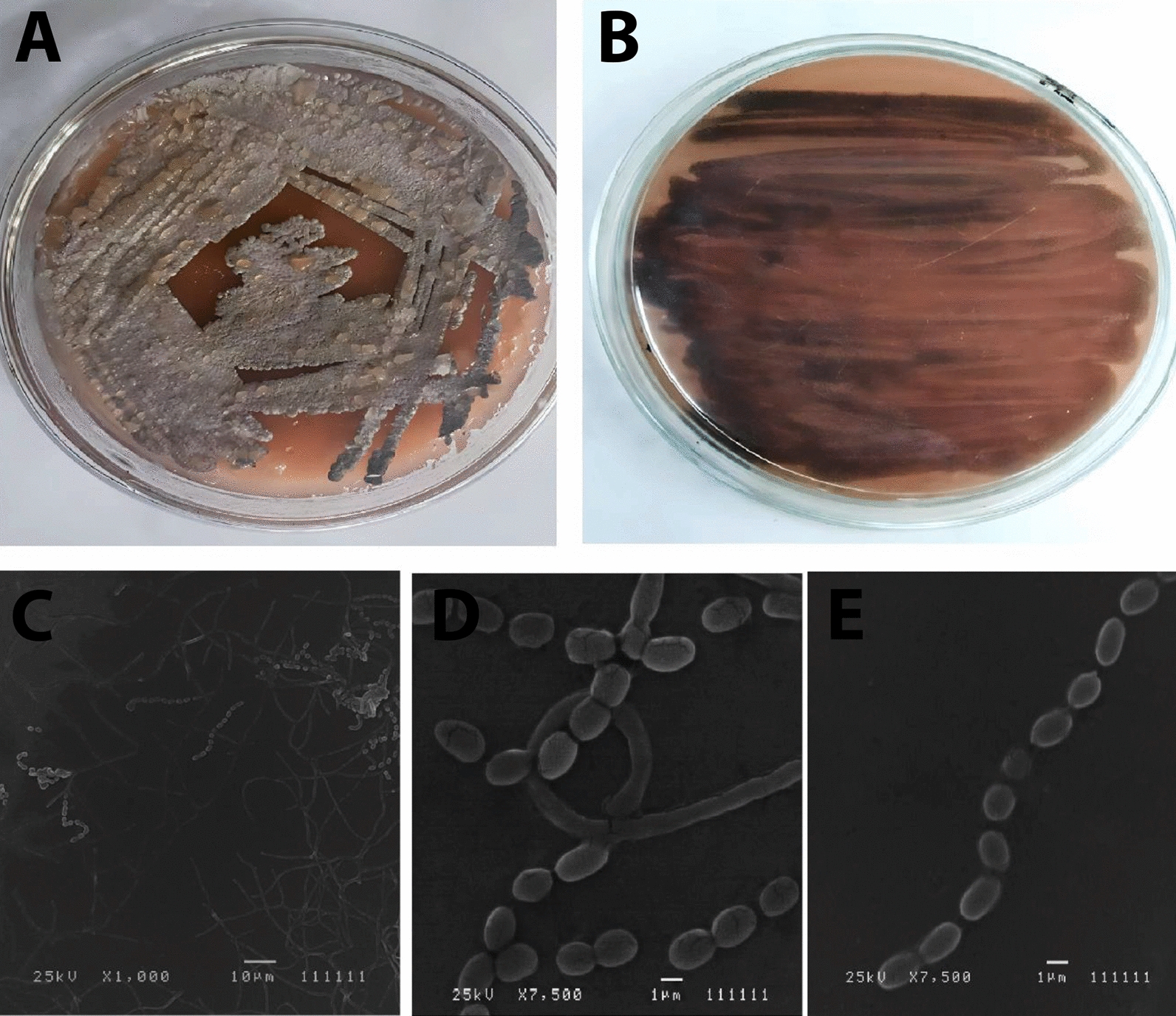
Morphological characteristics of ACT3 strain. Dusty aerial mycelium (**A**); Brown substrate mycelium (**B**); Scanning electron micrographs showing spore chain shape and spore surface ornamentation of ACT3 strain at different magnifications (1.000, 7.500, 7.500X) (**C**, **D**, **E**)

The morphological characteristics of the ACT3 strain, as shown in Fig. 2A, B, appear as dusty gray aerial mycelium with brown substrate mycelium colonies growing on ISP-6 medium. Moreover, scanning electron micrographs revealed that the selected isolate formed a straight to flexuous (rectiflexible) chain of globose spores with a smooth surface (Fig. 2C, D, and E). All these properties clearly suggest that the strain ACT3 belongs to the genus *Streptomyces*. Similar findings were done by Dastager, et al. [61], who showed that the ability to produce melanin was also found in 5–10% of *Streptomyces* isolates screened from soil samples in the Gulbarga region. Also, only two strains out of 25 isolates from Melia dubia fields in two different locations in Tamil Nadu were capable of producing melanin [62].

**Fig. 3:**
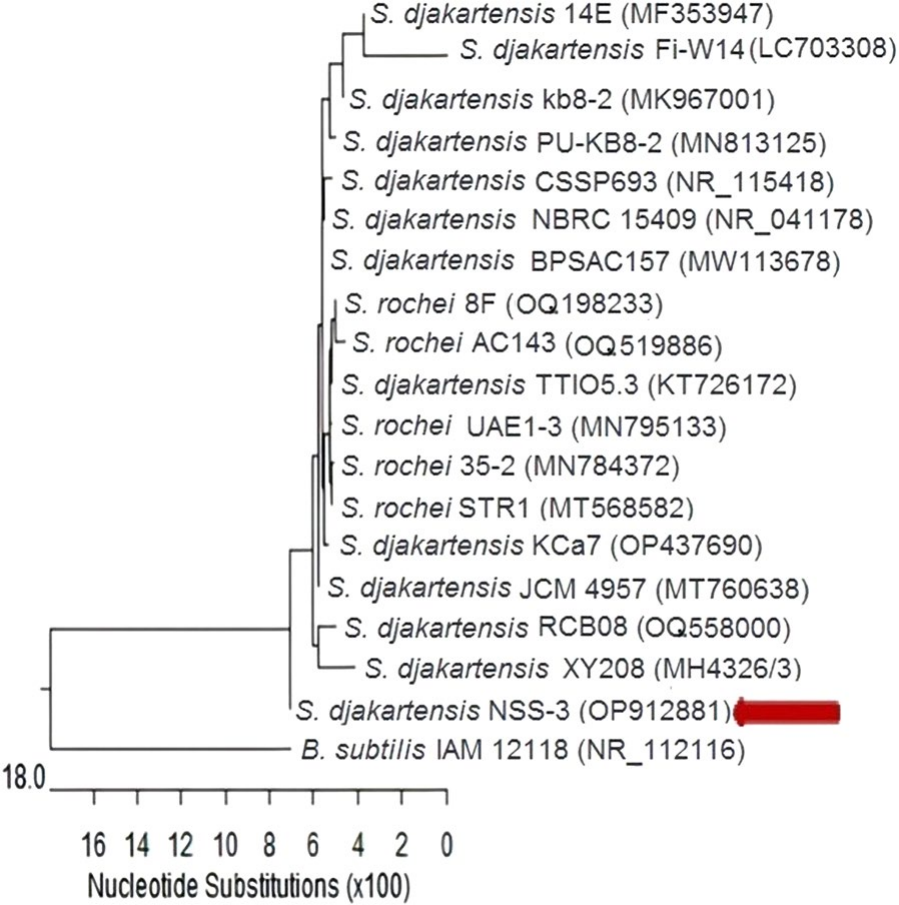
Phylogenetic tree based on 16S rDNA gene sequencing of *Streptomyces djakartensis* NSS-3 strain with GenBank accession no. OP912881 (arrowed) aligned with closely related sequences of bacterial strains accessed from the GenBank

Then, molecular identification by 16S rRNA confirmed ACT3 as *Streptomyces djakartensis* NSS-3 with accession number OP912881. *Streptomyces djakartensis* NSS-3 showed 99.87% similarity with *Streptomyces djakartensis* strain NBRC 15409 accession No. NR 041178.1 (Fig. 3). According to our knowledge, this is the first report showing *S. djakartensis* as a melanin-producing strain from soil.

### Melanin quantification and optimization

Because of the multiple and diverse factors that affect melanin biosynthesis, there is no universally applicable culture media or cultivation condition for growing melanogenic microorganisms. In our study, *S. djakartensis* NSS-3 produced melanin (28.38 mg/10 mL) after 7 days in PYI broth medium supplemented with tyrosine, similar to melanin produced by *Streptomyces glaucescens* strain NEAE-H (350 mg/L) on the same media [22]. While yeast melanin by *Yarrowia lipolytica* was 160 mg/L in M8 medium [63]. Media constituents and ratios of each component vary in microbial melanin production depending on the producing isolate [64]. Bacteria use melanogenesis via the DOPA pathway as a mean to combat the harmful effects of phenolic chemicals present in the environment, including those produced by bacteria during host defense [65]. Therefore, several microorganisms rely on external sources of tyrosine or tyrosine derivatives to carry out the process of melanin production. Scientists that research microbial melanization find this particularly interesting since melanin is produced extracellularly, which eliminates the need for aggressive extraction methods. Moreover, these attributes provide significant manipulation over the quantity and quality of the resultant melanin. While tyrosine is recognized as the primary substrate for melanin, other catechol amines, including dopamine and norepinephrine, may also serve as substrates. It is important to note that melanin produced from different sources might have varying structures because of distinct breakdown processes involving specific enzymes. This allows for the adjustment of the physicochemical characteristics and the enhancement of microbial melanin synthesis [66]. Similarly, environmental factors, i.e., temperature, pH, the presence of oxygen and aeration during cultivation, can greatly affect the cell growth and pigment biosynthesis, and should be carefully considered. Thus, applying statistical optimization methods can aid significantly in increasing the melanin yield by detecting the key parameters affecting the process and the effects of the interaction between those valuable parameters [67]. Optimization of the melanin production from *S. djakartensis* NSS-3 has been carried out using Plackett–Burman and Box-Behnken experimental designs.

**Fig. 4:**
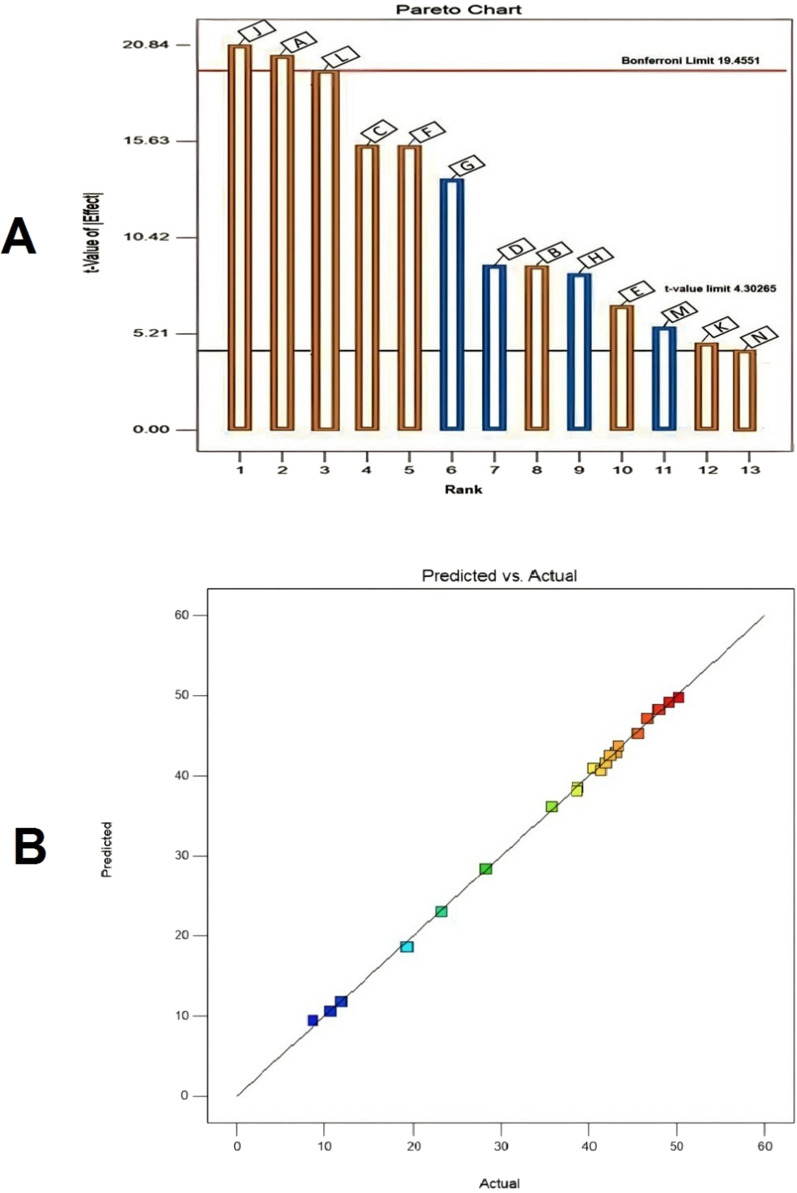
Pareto chart depicts the degree to which each variable influences melanin production by *S. djakartensis* NSS-3 (**A**); Correlation between the experimentally actual and predicted values for melanin production by *S. djakartensis* NSS-3 according to the Plackett–Burman experimental results (**B**)

In order to assess the variables that significantly affect melanin production, a PBD design was employed. Thirteen variables were examined, and their effects on melanin production were summarized in Table 1. PBD experimental results showed a wide variation in melanin production from 8.67 to 50.22 mg/10 mL of medium. The Pareto chart (Fig. 4A) indicated that L-tyrosine, incubation period, ferric ammonium citrate, temperature, yeast extract, pH, agitation speed, copper sulphate, and sodium thiosulphate exerted positive effects on melanin production (orange columns), whereas peptone, peptic digest, inoculum size, and dipotassium phosphate had a negative effect (blue columns). The predicted versus actual melanin production plot (Fig. 4B) confirmed the model's adequateness by showing that the experimental results agree closely with the theoretical values predicted by the model equation. The results of the response's statistical analysis are shown in Table 2. The P-value was used to determine how each component affected the production of melanin. The coefficient of determination R^2^ was used to assess the model's fitness. The R^2^ value was 0.9990, which indicates that the model explains 99% of the variability in the response. Therefore, in the current investigation, the model is a reliable estimator of melanin production. The adjusted determination coefficient's value (Adj.R^2^ = 0.9909) and the predicted R squared of 0.8508 are similarly quite high, indicating a high significance of the model. This indicated that the predicted and observed values were very closely aligned. The experimental design's analysis of variance (ANOVA) was calculated, and the model F-value of 122.51 implies the model is significant. The analysis showed that L-tyrosine (J) was determined to be the most significant factor affecting melanin production by *S. djakartensis* strain NSS-3 at 99.77% confidence, followed by incubation period (A) at 99.76% confidence, and ferric ammonium citrate (L) at 99.58% confidence. The design expert's regression equation is given by:

$$ \left( \begin{aligned}   {\text{Y }} = {\text{ }} & {\text{35}}.{\text{35 }} + {\text{ 1}}0.{\text{41 A }} + {\text{ 3}}.{\text{36 B }} - {\text{ 4}}.{\text{44 C }} \\     &  + {\text{ }}0.{\text{3818 D }} - {\text{ }}0.{\text{56}}0{\text{9 E }} + {\text{ 3}}.{\text{57 F }} + {\text{ 1}}.{\text{51 G }} \\     &  - {\text{2}}.{\text{86 H }} + {\text{ 8}}.{\text{29 J }} + {\text{ 1}}.{\text{59 K }} + {\text{ 5}}.{\text{45 L }} - {\text{ 1}}.{\text{97 M}} \\    {\text{ }} &  - {\text{ }}0.{\text{36}}0{\text{3 N }} + {\text{ 1}}0.{\text{53 AC }} - {\text{ 4}}.{\text{22 AD }} + {\text{ 2}}.{\text{54 CF }} - {\text{ 9}}.{\text{22 FG}} \\  \end{aligned}  \right) $$

where Y is the predicted melanin yield and A is the incubation period, B is pH, C is temperature, D is inoculum size, E is agitation speed, F is yeast extract, G is peptone, H is peptic digest, J is L-tyrosine, K is copper sulfate, L is ferric ammonium citrate, M is dipotassium phosphate, and N is sodium thiosulfate.

**Table 1:** The applied Plackett–Burman experimental design for the production of melanin pigment by *S. djakartensis* NSS-3

Run No	Coded levels of independent variables																			Melanin production (mg/10 mL)		Residuals
	A	B	C	D	E	F	G	H	J	K	L	M	N	O	P	Q	R	S	T	Actual value	Predicted value	
1	+ 1	− 1	− 1	+ 1	+ 1	+ 1	+ 1	− 1	+ 1	− 1	+ 1	− 1	− 1	− 1	− 1	1	1	− 1	1	42.38	42.57	− 0.1922
2	− 1	+ 1	− 1	− 1	− 1	− 1	+ 1	+ 1	− 1	+ 1	+ 1	− 1	− 1	1	1	1	1	− 1	1	46.63	47.16	− 0.5322
3	− 1	+ 1	− 1	+ 1	− 1	− 1	− 1	− 1	+ 1	+ 1	− 1	+ 1	+ 1	− 1	− 1	1	1	1	1	41.86	41.62	0.2439
4	− 1	− 1	− 1	− 1	− 1	− 1	− 1	− 1	− 1	− 1	− 1	− 1	− 1	− 1	− 1	− 1	− 1	− 1	− 1	10.70	10.60	0.0961
5	+ 1	− 1	+ 1	− 1	+ 1	− 1	− 1	− 1	− 1	+ 1	+ 1	− 1	+ 1	1	− 1	− 1	1	1	1	38.66	38.13	0.5322
6	− 1	− 1	+ 1	+ 1	− 1	+ 1	+ 1	− 1	− 1	+ 1	+ 1	+ 1	+ 1	− 1	1	− 1	1	− 1	− 1	8.67	9.45	− 0.7761
7	+ 1	+ 1	− 1	− 1	+ 1	+ 1	+ 1	+ 1	− 1	+ 1	− 1	+ 1	− 1	− 1	− 1	− 1	1	1	− 1	23.20	23.01	0.1922
8	− 1	− 1	− 1	+ 1	+ 1	− 1	+ 1	+ 1	− 1	− 1	+ 1	+ 1	+ 1	1	− 1	1	− 1	1	− 1	41.31	40.68	0.6283
9	− 1	+ 1	+ 1	− 1	− 1	+ 1	+ 1	+ 1	+ 1	− 1	+ 1	− 1	+ 1	− 1	− 1	-1	− 1	1	1	19.30	18.62	0.6799
10	+ 1	− 1	− 1	− 1	− 1	+ 1	+ 1	− 1	+ 1	+ 1	− 1	− 1	+ 1	1	1	1	− 1	1	− 1	43.02	42.92	0.0961
11	− 1	− 1	+ 1	+ 1	+ 1	+ 1	− 1	+ 1	− 1	+ 1	− 1	− 1	− 1	− 1	1	1	− 1	1	1	11.86	11.80	0.0516
12	+ 1	+ 1	− 1	+ 1	+ 1	− 1	− 1	+ 1	+ 1	+ 1	+ 1	− 1	+ 1	− 1	1	− 1	− 1	− 1	− 1	40.52	40.96	− 0.4361
13	− 1	+ 1	+ 1	− 1	+ 1	+ 1	− 1	− 1	+ 1	+ 1	+ 1	+ 1	− 1	1	− 1	1	− 1	− 1	− 1	38.75	38.56	0.1922
14	+ 1	− 1	+ 1	− 1	− 1	− 1	− 1	+ 1	+ 1	− 1	+ 1	+ 1	− 1	− 1	1	1	1	1	− 1	43.30	43.73	− 0.4361
15	+ 1	+ 1	− 1	+ 1	− 1	+ 1	− 1	− 1	− 1	− 1	+ 1	+ 1	− 1	1	1	− 1	− 1	1	1	45.63	45.29	0.3400
16	+ 1	+ 1	+ 1	− 1	+ 1	− 1	+ 1	− 1	− 1	− 1	ss− 1	+ 1	+ 1	− 1	1	1	− 1	− 1	1	47.90	48.29	− 0.3845
17	+ 1	+ 1	+ 1	+ 1	− 1	+ 1	− 1	+ 1	− 1	− 1	− 1	− 1	+ 1	1	− 1	1	1	− 1	− 1	49.04	49.19	− 0.1477
18	− 1	− 1	− 1	− 1	+ 1	+ 1	− 1	+ 1	+ 1	− 1	− 1	+ 1	+ 1	1	1	− 1	1	− 1	1	35.75	36.19	− 0.4361
19	+ 1	− 1	+ 1	+ 1	− 1	− 1	+ 1	+ 1	+ 1	+ 1	− 1	+ 1	− 1	1	− 1	− 1	− 1	− 1	1	50.22	49.78	0.4361
20	− 1	+ 1	+ 1	+ 1	+ 1	− 1	+ 1	− 1	+ 1	− 1	− 1	+ 1	− 1	1	1	− 1	1	1	− 1	28.28	28.43	− 0.1477

A (incubation period), B (pH), C (temperature), D (inoculum size), E (agitation speed), F (yeast extract), G (peptone), H (peptic digest), J (L-tyrosine), K (copper sulfate), L (ferric ammonium citrate), M (dipotassium phosphate), and N (sodium thiosulphate)

**Table 2:** Analysis of variance (ANOVA) and regression statistics for the experimental results of Plackett Burman design of melanin production by *S. djakartensis* NSS-3

Source	SS	df	MS	F-value	P-value	Confidence level (%)
Model	3420.80	17	201.22	122.51	0.0081*	99.19
Incubation period (A)	674.99	1	674.99	410.94	0.0024*	99.76
pH (B)	129.51	1	129.51	78.85	0.0124*	98.76
Temperature (C)	303.13	1	303.13	184.55	0.0054*	99.46
Inoculum size (D)	1.69	1	1.69	1.03	0.4174*	58.26
Agitation speed (E)	4.12	1	4.12	2.51	0.2541*	74.59
Yeast extract (F)	74.58	1	74.58	45.40	0.0213*	97.87
Peptone (G)	34.02	1	34.02	20.71	0.0450*	95.5
Peptic digest (H)	117.63	1	117.63	71.62	0.0137*	98.63
L-tyrosine (J)	713.46	1	713.46	434.37	0.0023*	99.77
Copper sulphate (K)	36.45	1	36.45	22.19	0.0422*	95.78
Ferric ammonium citrate (L)	389.38	1	389.38	237.06	0.0042*	99.58
Dipotassium phosphate (M)	51.08	1	51.08	31.10	0.0307*	96.93
Sodium thiosulphate (N)	1.49	1	1.49	0.9056	0.4417*	55.83
Residual	3.29	2	1.64			
Cor Total	3424.09	19	R-Squared			0.9990
Std. Dev	1.28					
Mean	35.35		Adjusted R-Squared			0.9909
C.V. %	3.63		Predicted R-Squared			0.8508
			Adeq Precision			33.1686

*Significant values, *SS* sum of squares, *MS* mean of square, *F* Fishers, s function, P: Level of significance, CV %- the coefficient of variation %

**Fig. 5:**
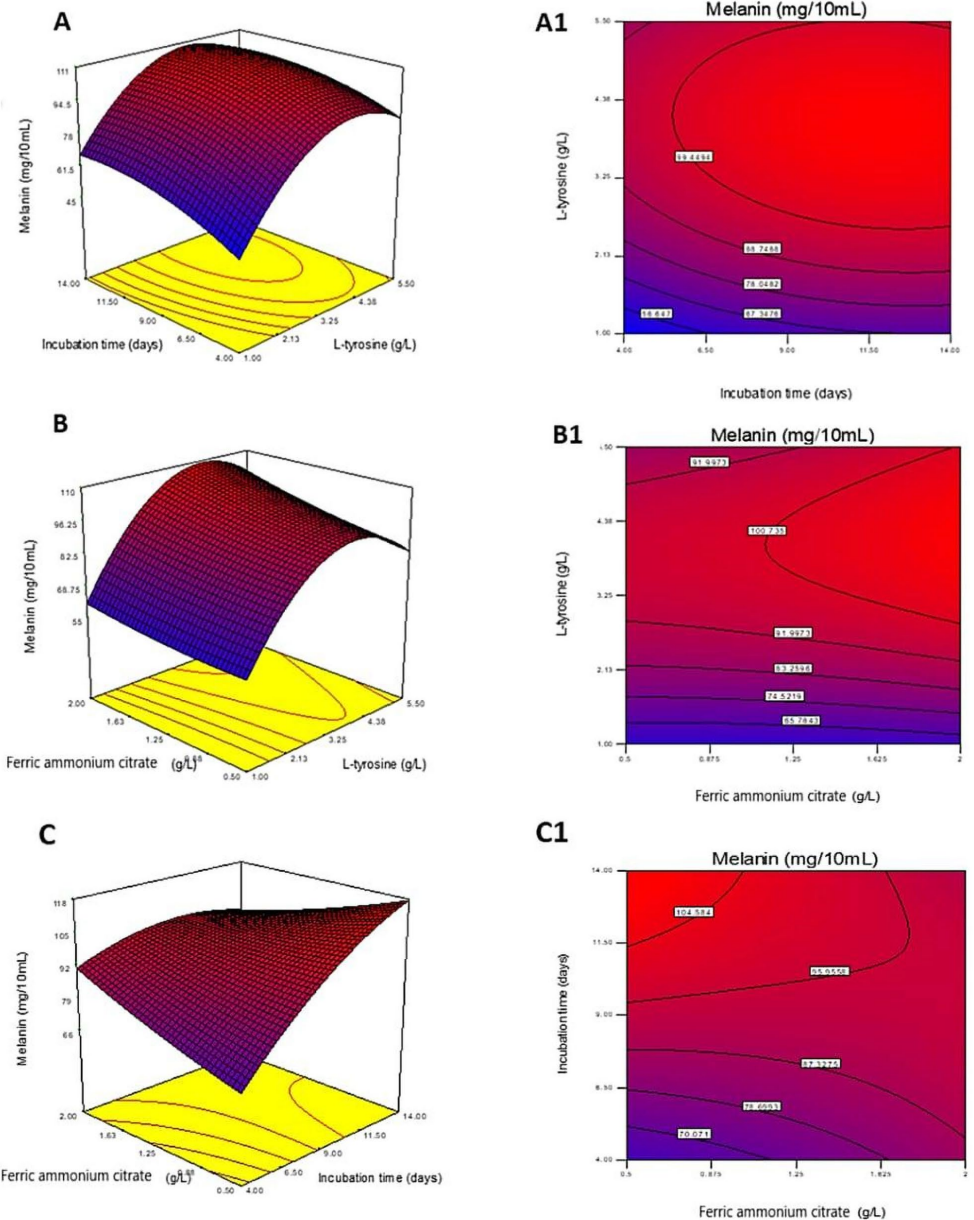
The 3D surface response and contour plots showing the effect of L-tyrosine (**A**, **A1**); incubation period (**B**, **B1**); and ferric ammonium citrate (**C**, **C1**) and their mutual interaction on melanin production

**Fig. 6:**
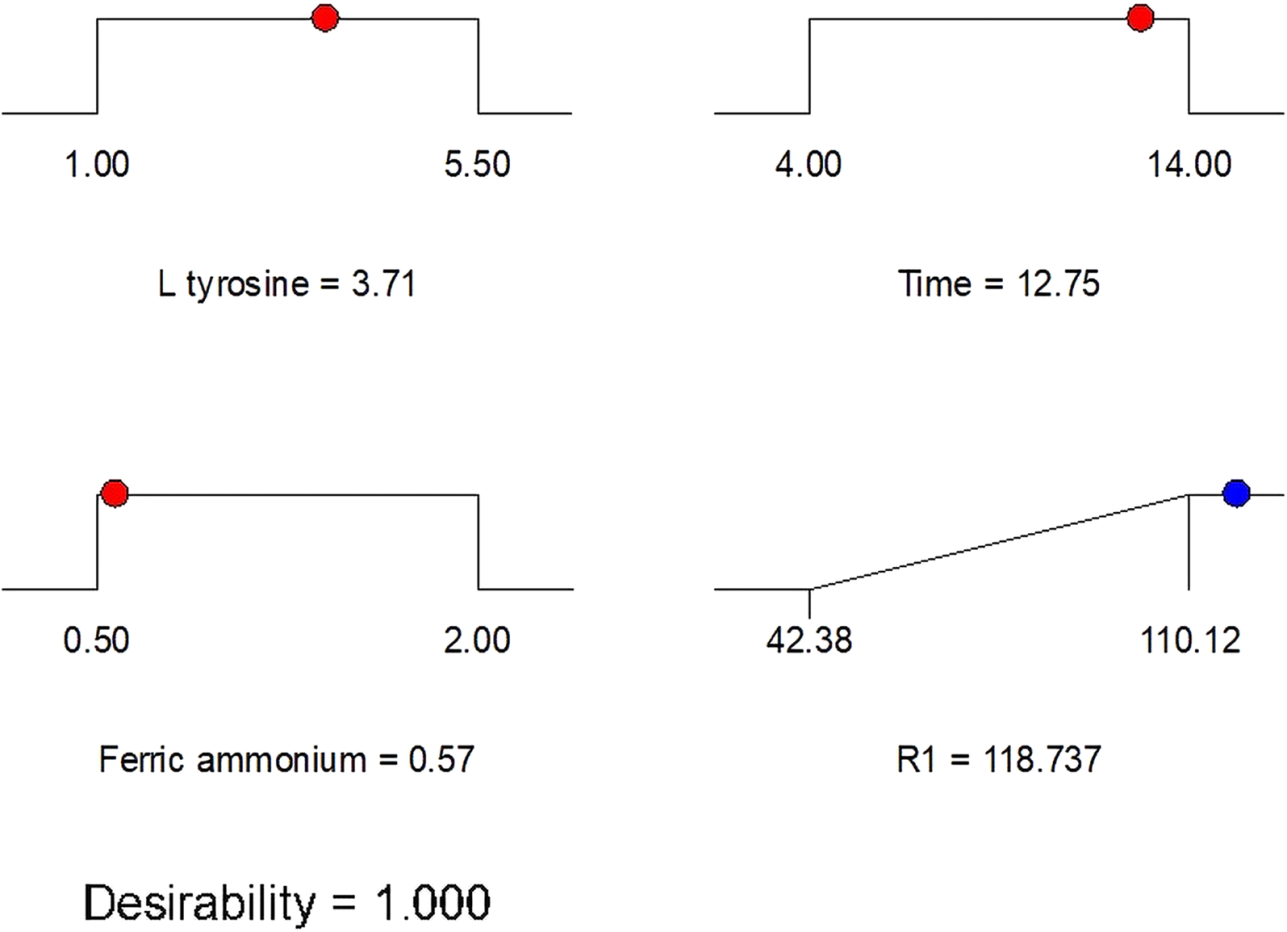
Predicted solution for maximum melanin pigment production using BBD numerical optimization

**Fig. 7:**
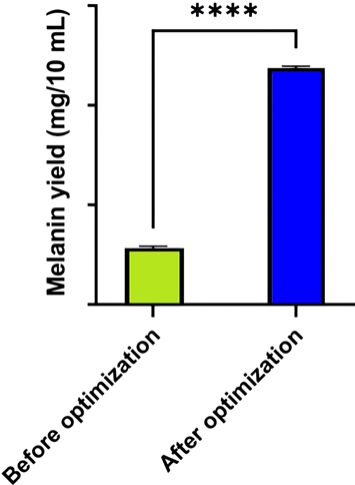
Melanin production before and after RSM optimization

Based on the results of PBD, the most effective parameters affecting melanin production are L-tyrosine, incubation period, and ferric ammonium citrate, which were selected for further optimization using RSM with BBD. Response surface methodology was employed in this work to detect the levels required for the applied parameters to produce the desired value of the response in a limited number of tests [68]. The interactions between the different parameters have also been evaluated using this method [69]. Many researchers have found that the Box-Behnken design method of RSM is a reliable and effective tool for the optimization and formulation of a wide range of processes [70]. The BB experiment was designed and conducted using the most effective parameters, as shown in Table 3. The results obtained were submitted to ANOVA using the Design Expert software. A t-test analysis of the polynomial model's statistical significance yielded the data presented in Table 4. The polynomial model was found to be statistically significant (p = 0.0378) in an analysis of variance (ANOVA). The coefficient of determination (R^2^ = 0.8410) suggested that the variation in melanin yield could be attributed to medium components, which was consistent with the data. Furthermore, A and B have been reported as significant model terms. A significant value (0.0014) indicated a lack of fit. Our findings indicated that the model's quality was satisfactory, suggesting that it could accurately describe the relationship between the various medium components. Moreover, it has been reported that A and B are significant model terms. The lack of fit value was significant (0.0014). Results showed that the quality of the model was adequate and might describe the real relationship among medium components. The quadratic polynomial equation estimated by the model regression analysis was as follows:

$$ {\text{R1}} = { 103.44} + {16.26 }*{\text{ A }} + 13.80 \, *{\text{ B }} + {1.80} \, *{\text{ C }} - {2.17}*{\text{ A }}*{\text{ B }} + 3.00 \, *{\text{ A }}*{\text{ C }} - {11.21 }*{\text{ B }}*{\text{ C }} - {24.04 }*{\text{ A}}^{{2}} - {6.44 }*{\text{ B}}^{{2}} + {1.46 }*{\text{ C}}^{{2}} $$

where R1 is the melanin yield, A is the coded value for L-tyrosine, B is the coded value for the incubation period, and C is the coded value for ferric ammonium citrate. The main effects of independent variables on melanin production and their interactions were represented using a three-dimensional response surface and contour plots. The 3D response surface plots and contour plots for significant pair-wise combinations of the three variables (AB, AC, and BC) are presented in Fig. 5. The melanin yield was plotted on the z-axis of each three-dimensional response surface plot (Fig. 5A, B, and C) against two independent variables that were investigated simultaneously, while the third variable was held constant at zero (intermediate value). There was an insignificant interaction between the tested variables. The correlation between each of the two variables does not help much in increasing melanin production [71]. It was observed that the increase in L-tyrosine concentration and incubation period value led to an increase in melanin production. While an increase in ferric ammonium citrate led to a decrease in melanin production.With the aid of BBD numerical optimization, the optimal conditions for melanin production were determined to be 3.71 g/L L-tyrosine, 12.75 days of incubation, and 0.57 g/L ferric ammonium citrate (Fig. 6). After applying the numerical optimization design, the yield of melanin increased 4.19-fold as compared to the yield before the entire optimization step (Fig. 7). A similar study used an identical approach to maximize *S. glaucescens* NEAE-H productivity of melanin [22]. Results showed that 31.65 g melanin/0.1 mL of medium could be obtained with an incubation time of 6 days, a protease-peptone concentration of 5 g/L, and a ferric ammonium citrate concentration of 0.5 g/L.

**Table 3:** Seventeen trials of Box-Behnken design representing melanin production by *S. djakartensis* NSS-3

Run No	Experimental parameters			Melanin yield (mg/10 mL)	
	A	B	C	Actual value	Predicted value
1	− 1	1	0	42.38	40.73
2	0	− 1	− 1	60.75	77.59
3	1	0	− 1	89.51	72.67
4	0	1	− 1	99.20	100.85
5	0	1	1	60.50	65.80
6	0	0	0	105.50	92.32
7	0	− 1	1	50.22	63.40
8	1	− 1	0	107.22	101.92
9	0	0	0	75.30	71.65
10	0	0	0	110.12	121.66
11	0	0	0	109.20	97.66
12	1	0	1	99.20	102.85
13	0	0	0	105.20	103.44
14	− 1	− 1	0	102.40	103.44
15	1	1	0	98.75	103.44
16	− 1	0	− 1	106.33	103.44
17	− 1	0	1	104.50	103.44

**Table 4:** ANOVA of the fitted quadratic polynomial model of melanin production by *S. djakartensis* NSS-3

Source	Sum of Squares	df	Mean square	F value	P-value Prob > F	
Model	6904.38	8	767.15	4.12	0.0378	Significant
A-L tyrosine	2114.45	1	2114.45	11.34	0.0120	
B-Time	1523.52	1	1523.52	8.17	0.0244	
C-Ferric ammonium citrate	25.99	1	25.99	0.14	0.7199	
BC	18.84	1	18.84	0.10	0.7599	
A2	36.00	1	36.00	0.19	0.6736	
B2	502.21	1	502.21	2.69	0.1447	
C2	2432.44	1	2432.44	13.05	0.0086	
BC2	174.65	1	174.65	0.94	0.3653	
Residual	8.97	8	8.97	0.048	0.8326	
Lack of Fit	1304.86	4	186.41	47.47	0.0014	Significant
Pure Error	1269.21	4	423.07			
Cor Total	35.65	16	R2 = 0.8410

In our study, L-tyrosine had a significant effect on melanin production by *S. djakartensis* NSS-3, as it is considered a precursor for melanin synthesis. A similar study showed that *Bacillus safensis* is capable of synthesizing approximately 6.69 g/L of melanin when the culture medium is supplemented with tyrosine [72]. Our findings are similar to those of Quadri and Agsar [73], who found that thermo-alkaliphilic *Streptomyces* produced the most melanin when given tyrosine as a simple nitrogen source. In contrast, marine *Pseudomonas stutzeri* isolated from seaweed was shown to produce considerable levels of melanin, with a melanin concentration of 6.7 g/L after 10 h of incubation without tyrosine supplementation [74]. According to our findings, the optimal time for producing melanin was the 13th day of incubation. This finding corroborated the work of Babitskaya, et al. [75], who found that the optimal time to extract melanin pigment from *Aspergillus carbonicus* was between the 15th and 25th days of incubation. Ferric ammonium citrate has a positive effect on melanin production by *S. djakartensis* NSS-3 as the organism reduces ferric citrate by ferric reductase, converting ferric into ferrous, and these ferrous metal ions are critical for melanin production. Wang, et al. [76] reported that iron supplementation promoted the generation of melanin by inducing the synthesis of tyrosinase. For model validation, the model was verified by culturing the strain *S. djakartensis* NSS-3 in the predicted optimum medium obtained from BBD. The maximum yield for melanin (118.737 mg/10 mL) obtained from model verification experiments was found to be very close to the predicted response (115.89 mg/10 mL), giving a deviation of 2.8%, suggesting the high accuracy of the model. As a result, BB design was found to be an accurate and decisive tool for predicting the extraction yield of melanin from *S. djakartensis* NSS-3. According to the previously illustrated data, it can be expected that in order to achieve the highest production of melanin pigment by *S. djakartensis* NSS-3, the formula of the medium should be as follows (g/L): yeast extract (1.5 g/L); peptone (3 g/L); peptic digest (10 g/L); L-tyrosine (3.71 g/L); copper sulfate (0.1 g/L); ferric ammonium citrate (0.57 g/L); dipotassium phosphate (0.5 g/L); sodium thiosulfate (0.1 g/L); inoculum size, 1 mL; pH 7; incubation period of 13 days at 40 °C with an agitation speed of 200 rpm.

### Extraction and physiochemical properties of melanin

Purified melanin pigment is separated using the TLC technique after standardizing the solvent proportions to a 4:4:6:1 ratio of petroleum ether: ethyl acetate: 95% ethanol: conc. ammonia, respectively. A chromatogram was generated, and separated spots of pigment were observed in the UV lamp. Retention factor (R_f_) value was calculated to be 0.8. The separated bands revealed an equal R_f _value with standard synthetic melanin as in Additional file 1: Fig. S2, similar to the TLC analysis of melanin done by Diraviyam, et al. [77]. As a first step in identifying and characterizing the purified melanin, traditional physicochemical tests could be used because of melanin's rare solubility and reactivity. The chemical characterization of the dark brown melanin pigment extracted from *S. djakartensis* NSS-3 is presented in Additional file 1: Table S5. The purified brown powder was found to be partially soluble in water as well as in absolute ethanol, methanol, chloroform, acetone, benzene, and ethyl acetate. After vigorous shaking, the purified pigment was found to be soluble in dimethyl sulfoxide (DMSO), potassium hydroxide, and sodium hydroxide (1N) solutions. The temperature tolerance and stability of purified melanin were tested, and it was extremely resistant to temperature. Visible peaks in the UV spectrophotometer at 255 nm indicated temperature stability. Additionally, the extracted melanin precipitated when exposed to a solution of 3N HCl and 1% ferric chloride. Hydrogen peroxide (30% v/v) was used to remove the color, and KMnO_4_ was added to transform the brown pigment into a clear solution. Physicochemical comparisons of the standard synthetic melanin and the purified melanin are identical, as shown in Additional file 1: Table S5, similar to melanin reported from other microorganisms [78–80] and characterized by its dark brown color and is partially soluble in water and the majority of organic and inorganic solvents. Our results are consistent with those of Kamarudheen, et al. [81], who found that the extracellular melanin pigment produced by marine *Nocardiopsis* spp. was insoluble in ethyl acetate and chloroform but soluble in dimethyl sulfoxide and alkaline water (pH = 10).

### Characterization of melanin

**Fig. 8:**
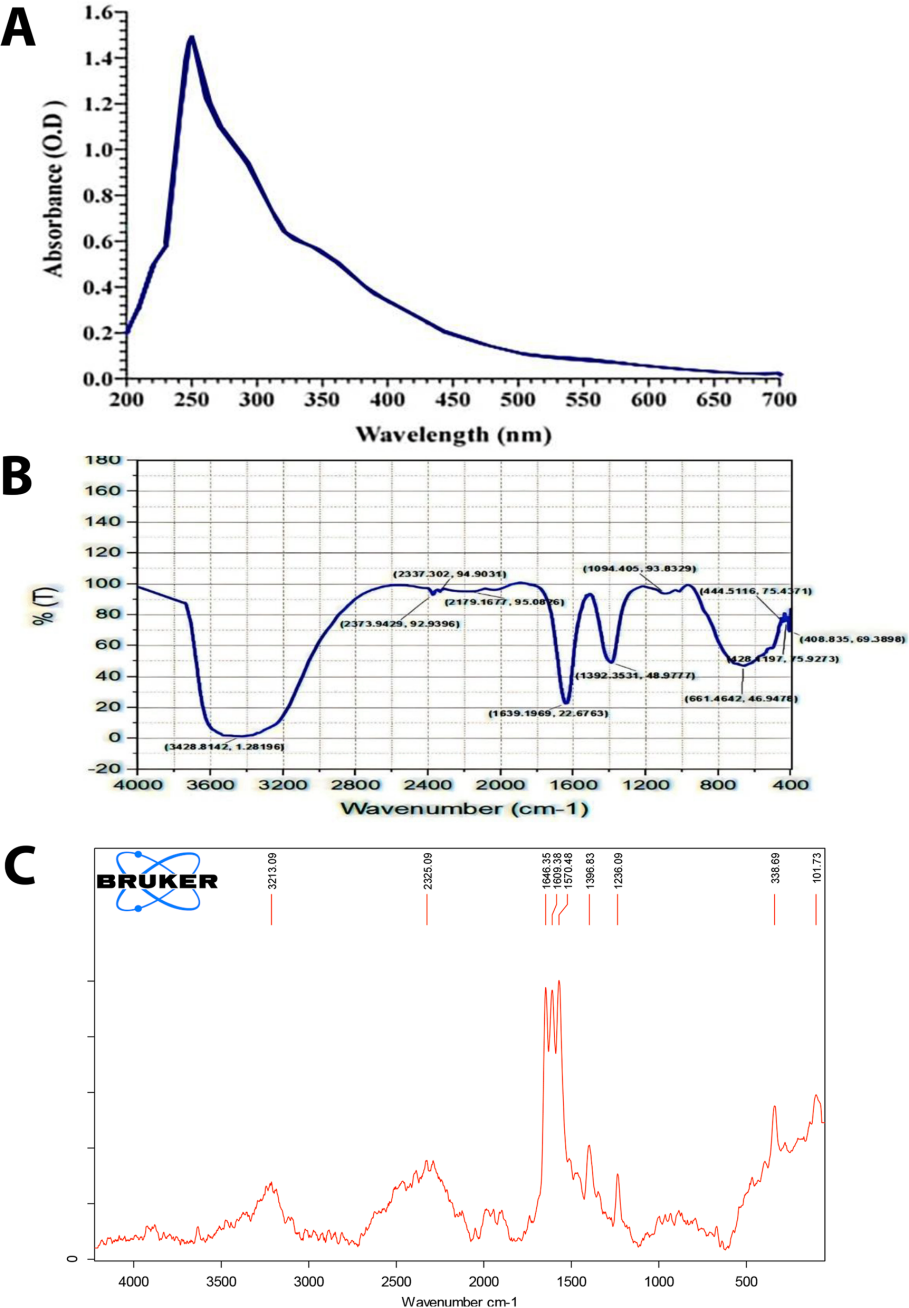
Spectroscopic analysis of the purified melanin produced by *S. djakartensis* NSS-3. Uv–visible spectrum (**A**); FTIR spectroscopic spectrum (**B**); Raman spectrum (**C**)

As shown in Fig. 8A, the maximum absorption wavelength of the UV–vis spectrum of purified melanin produced by *S. djakartensis* NSS-3 was 255 nm, and its optical density gradually decreased as the wavelength increased toward the visible wavelength, revealing the typical nature of the melanin absorbance. This result was similar to the previous study of Dadachova, et al. [82], who revealed that the melanin molecule might contain a conjugated double bond system or an aromatic ring structure. Furthermore, there was no absorption peak at 260 or 280 nm, indicating that melanin did not contain nucleic acids or proteins [83]. When compared to standard melanin [84], melanin pigment extracted from *Cryptococcus rajasthanensis* KY627764 showed similar results, with absorbance peaks at 244 and 220 nm. In addition, our spectral data were consistent with those published by Elsayis, et al. [85], who found that the absorbance maxima of UV spectral data of extracted melanin from *Hortaea werneckii* AS1 was at 255 nm.

The FT-IR spectrum can be used to identify melanin because it provides details about the structure's primary functional groups [86]. Melanin is a complex macromolecule with a characteristic infrared spectrum featuring a series of broad, intense absorption peaks. Each broad peak is generated by a large number of functional groups [87]. The FTIR spectra of purified melanin pigment derived from S. *djakartensis* NSS.3 (Fig. 8B) showed a number of significant peaks that suggest the following peak assignments: OH group (3442.31 cm^−1^), CH_2_ stretching (2372.01 cm^−1^), C = O or aromatic stretching (1645.95 cm^−1^), ionized COO^−^ groups (1397.17 cm^−1^), combination of C-O bonds (1105.01 cm^−1^) and weak bands below 700 cm^−1^ represent the alkene C-H replacement in melanin. These FTIR features are identical with those of pyomelanin produced by different microorganisms [88–90]. Moreover, the molecular vibration and crystal structures of the purified melanin were analyzed using Raman spectroscopy. The Raman spectra of purified melanin in the range of 500–5000 cm^−1^ are shown in Fig. 8C. The recorded spectra displayed multiple bands between 1000 and 1800 cm^−1^; the 1236.09 cm^−1^ band corresponds to the phenolic C–OH stretching vibration and the carboxylic acid C–O stretching vibration, while the 1396.83 cm^−1^ band corresponds to the aromatic C–C linear stretching vibration. Two bands at 1570.48 cm^−1^ and 1609.38 cm^−1^ can be attributed to the stretching in C = C [91]. Moreover, these results confirm that melanin derived from *Streptomyces djakartensis NSS-3* closely resembles the RAMAN spectra results of pyomelanin from *Aspergillus fumigatus*, as RAMAN bands at 1381 cm^−1^ and 1583 cm^−1^ served as a spectral signature for pyomelanin [92].

**Fig. 9:**
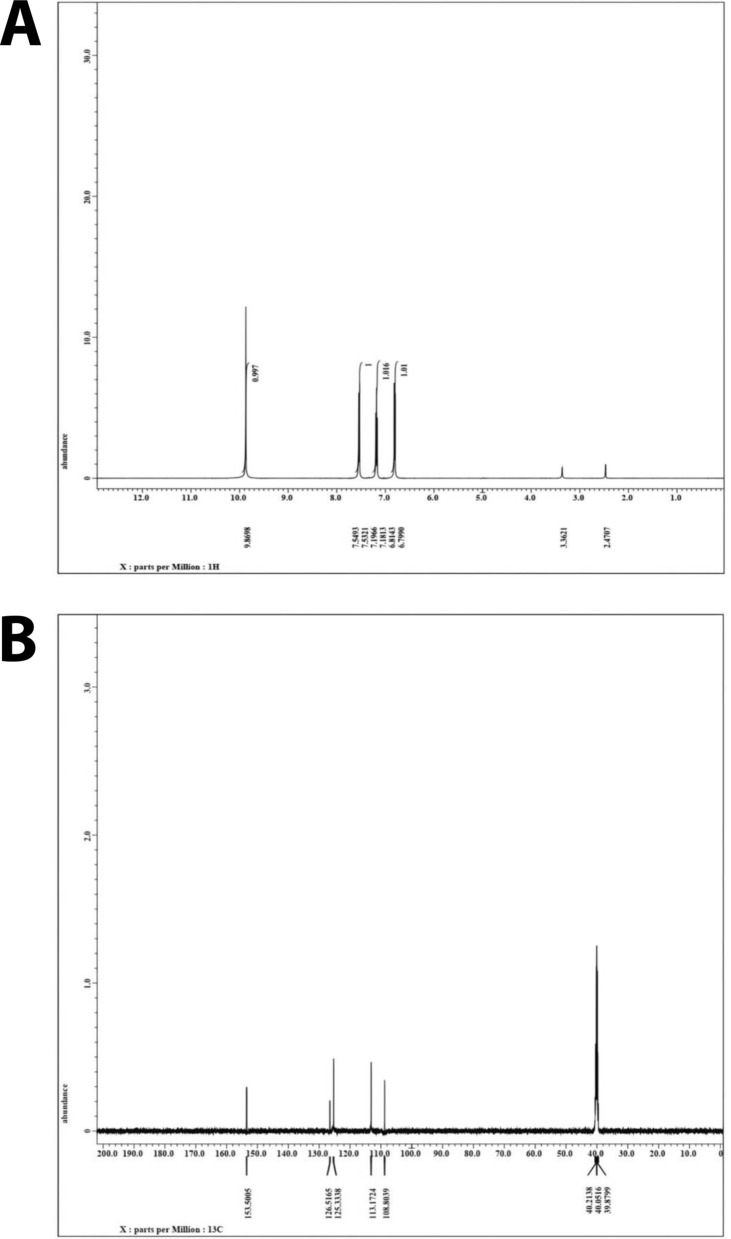
^1^HNMR (**A**); and.^13^CNMR (**B**) of purified melanin produced by *S. djakartensis* NSS-3

Furthermore, ^1^H and ^13^C-NMR spectra of purified melanin contain a number of chemical shifts that can be used to confirm the molecular structure of melanin. ^1^H NMR spectral analysis of the purified melanin pigment synthesized by *S. djakartensis* NSS-3 has shown resonances in both aliphatic and aromatic regions (Fig. 9A). The peak at 2.47 ppm indicated C14-H and C23-H [93, 94], while the peak centered at 3.36 ppm has been assigned to the presence of methyl or methylene groups attached to oxygen atoms [95], as has been found in other melanin pigments [93, 94, 96]. The resonances between 6.79 and 9.8 ppm in NMR spectra have been assigned to protons attached to the substituted aromatic and hetero-aromatic regions [97]. In the ^13^C-NMR spectra, the peaks between 120 and 140 ppm could be generated by protonated and non-protonated aromatic carbons. The resonance peaks of mostly protonated aliphatic carbons were detected within 10–40 ppm (Fig. 9B). The results obtained in the present study corroborated the observations made earlier by Chatterjee, et al. [98] and Prados-Rosales, et al. [99]. Based on these findings, we suspect that the resultant pigment may be pyomelanin and not eumelanin, which lacks nitrogen. Similar results have been shown by Hocquet et al. [100] and Mahmood et al. [101].

**Fig. 10:**
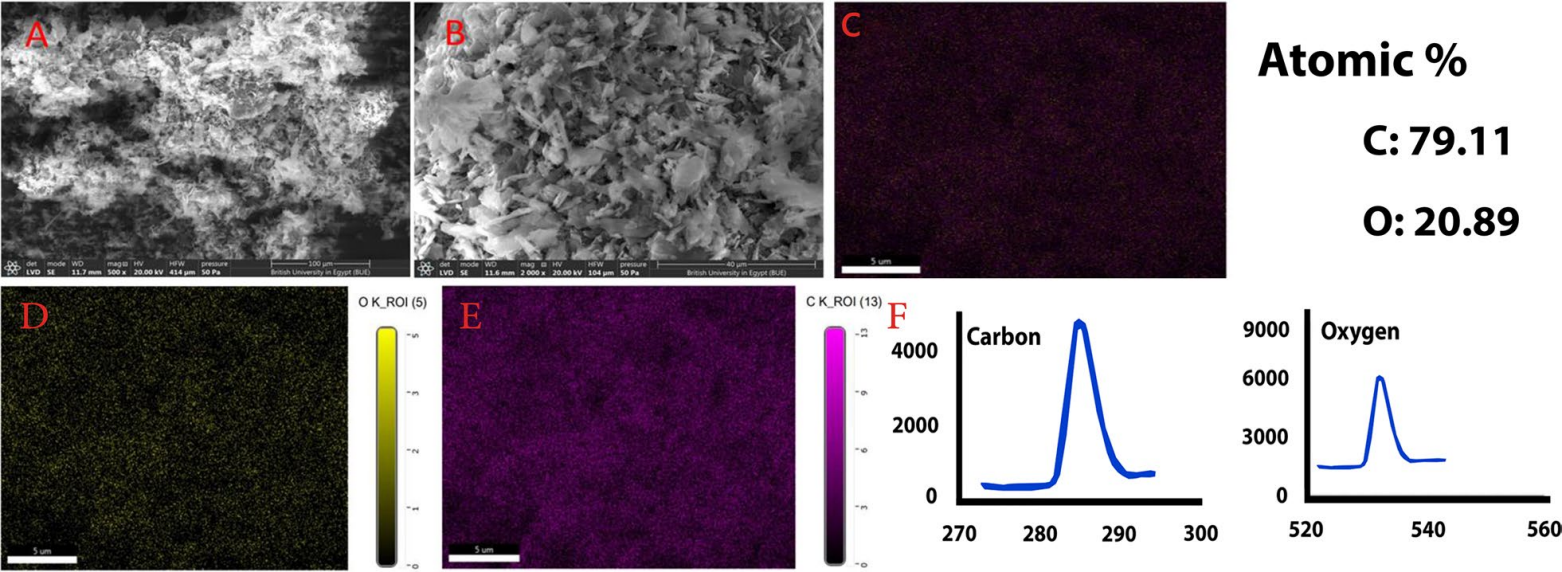
SEM images of purified melanin produced by *S. djakartensis* NSS-3 at different magnifications (**A**, **B**); Energy dispersive spectroscopy (EDS) and elemental mapping analysis showing elemental composition of purified melanin (**C**, **D**, **E**, **F**)

In the present study, SEM micrographs of purified melanin isolated from *S. djakartensis* NSS-3 are shown in Fig. 10A, B. Purified melanin appeared as aggregates with an amorphous (irregular) shape pattern, similar to past reports of purified bacterial melanin [102]. However, melanin obtained from *Streptomyces glaucescens* NEAE-H has been found to be small and sphere-shaped [22]. Further to knowing the compositional pattern, energy dispersive X-ray (EDX) spectroscopy analysis revealed that the main elements detected on the surface of the purified melanin were carbon and oxygen (Fig. 10F), which are considered the key elements in melanin structure [67]. Carbon and oxygen are present by atomic percentage (79.11 and 20.89%, respectively) in the absence of nitrogen. The elemental map of purified melanin pigment produced by *S. djakartensis* NSS-3 was demonstrated in Fig. 10C, D and E. The absence of nitrogen serves as additional support, which reflects that the extracted pigment may be pyomelanin [103]. Many strains, including *Pseudomonas aeruginosa* and *Legionella pneumophila* [104, 105], produce pyomelanin during the catabolism of tyrosine or phenylalanine via the oxidation of Homogentistic acid (HGA), which is consistent with our findings that to produce pyomelanin, HGA must undergo auto-oxidation and self-polymerization, which requires the involvement of 4-hydroxyphenylpyruvic acid dioxygenase (4-HPPD) and HGA-oxidase [106, 107].

### Sunscreen protection factor (SPF)

Melanin has developed commercial applications as a UV protector in cosmetics and food [108]. The UV-absorption and photostability of melanin are significantly higher than those of previously reported metabolites of palythine and mycosporine-like amino acids [109]. Melanin can protect the body from the sun because of its physiological and photoprotective properties. Melanin is resistant to breakdown and may eliminate as much as 90% of the heat generated by exposure to sunlight [110]. Currently, melanin is primarily used as a dye in the lenses of sunglasses. In this case, the advantage is particularly highlighted because of the pigment's natural origin and its ability to reduce high-energy visible light. In our study, the high SPF value of the purified melanin from *S. djakartensis* NSS-3 was determined to be 18.5 (Table 5), suggesting that it may be effective in protecting the skin from the harmful effects of UV rays when used as a sunscreen. Sunscreens with an SPF of 15 are recommended by the FDA to protect against sunburn, skin cancer, and premature aging of the skin [111]. Therefore, purified melanin from *S. djakartensis* NSS-3 is promising for future formulations of sunscreens and personal care products and it has fewer side effects compared to organic sunscreens [112, 113].

**Table 5:** SPF result of extracted melanin from *S. djakartensis* NSS-3

Wavelength (nm)	Abs. EE(λ) × I(λ)
290	0.0150
310	0.1964
315	0.0939
320	0.0180
Sample	SPF value
Extracted melanin	18.5

*EE* erythema effect spectrum, *I* solar intensity spectrum, *Abs* the absorbance of sunscreen product

Melanin has phenolic hydroxyl and carboxyl active groups, enabling it to absorb UV light and function as a physical barrier that disperses UVR. Additionally, it acts as an absorbent filter that diminishes the penetration of UV radiation through the epidermis [114]. Studies have shown that melanin interacts with DNA and functions as a photosensitizer, generating reactive oxygen species (ROS) upon exposure to UVA radiation [115]. In addition, melanin also enhances the secretion of histamine, which has a role in the development of sun-induced redness and swelling in people with fair skin [116].

### Antioxidants and cytotoxicity

**Fig. 11:**
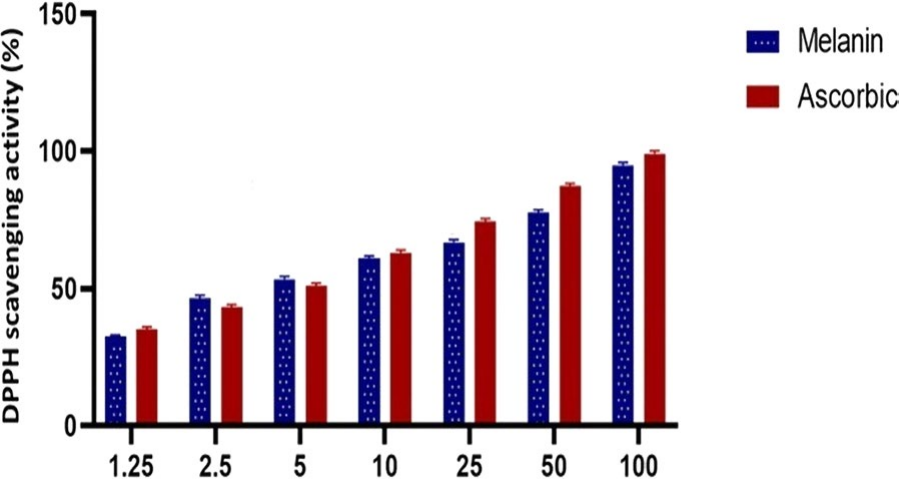
Antioxidant activity of purified melanin and ascorbic acid as standard

To determine the possible biomedical applications of purified melanin, we examined its potential antioxidant and anticancer activities. Antioxidants protect cells from free radicals and have a protective function for DNA and many other biologically important compounds [117]. Purified melanin pigment from *S. djakartensis* NSS-3 was tested for antioxidant activity against DPPH free radicals. A deep violet DPPH solution can be used to measure radical concentration because its color changes from deep violet to pale yellow or even colorless upon adding extracted melanin pigment [118]. As the concentration of extracted melanin pigment increased, so increased their DPPH activity, indicating a dose-dependent behavior. It exhibited scavenging activity of 32.2–94.82% at concentrations of 1.25–100 µg/mL, with an average IC_50_ value of 18.03 µg/mL as shown in Fig. 11. Purified melanin pigment has comparable antioxidant activity with standard ascorbic acid (IC_50_ = 16.83 µg/mL). The results obtained were similar to earlier studies, which also revealed that the antioxidant activity of melanin is dose-dependent. Rao and Rao [119] found free radical scavenging activity in the range of 56.58–68.91%, while Arun et al. [120] found it to be in the range of 87–96%. Melanin is a polymer with molecules that contain unpaired electrons and can either donate or accept an electron. Melanin pigment has many unpaired valence electrons, making it a great scavenger for free radicals and other reactive oxygen species. Melanin is an antioxidant that fights free radicals through a series of one-electron transfer reactions, suggesting its use in cosmetic products that minimize toxin-induced tissue damage [121]. Moreover, melanin has a strong affinity and great capacity for direct binding with metal ions like Fe^2+^ [122]. The presence of aromatic components and functional groups in melanin, including hydroxyl, carboxyl, amine, and phenol groups, allows melanin to chemically interact with many organic and inorganic compounds [123].

**Fig. 12:**
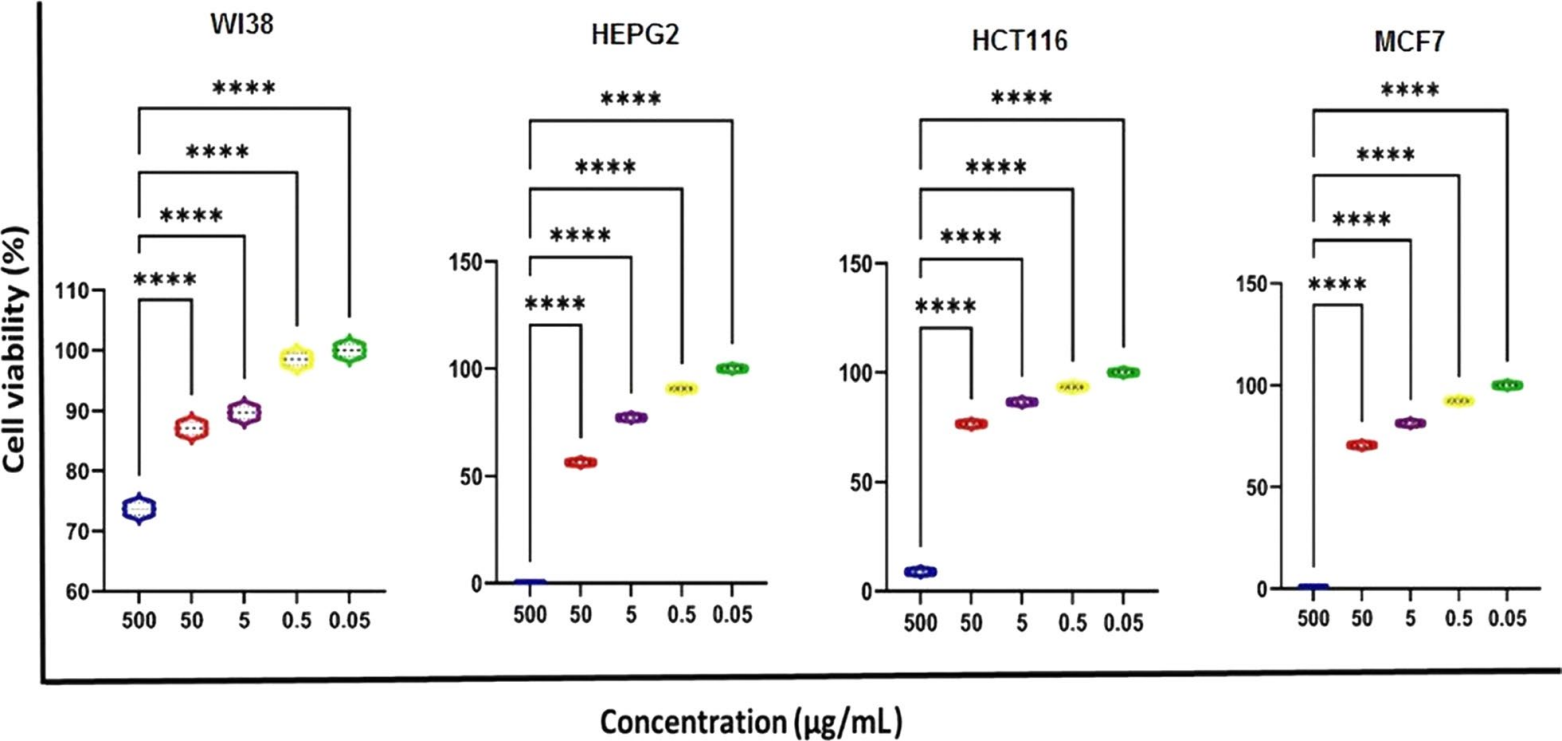
In vitro cytotoxicity and anticancer activities of various concentrations of the purified melanin pigment of *S. djakartensis* NSS-3

Furthermore, cytotoxicity evaluation is an important part of developing safe and effective medicines [124, 125]. In this study, we used in vitro models of WI38, HCT116, HEPG, and MCF7 cell lines to test the cytotoxicity of melanin using the MTT assay. Figure 12 depicted the cytotoxic effects of purified melanin against cancerous and noncancerous cell lines. The results showed that purified melanin was found to be non-toxic toward the WI38 cell line, permitting normal cell metabolism and growth (IC_50_ greater than 500 μg/mL). These results confirmed the high safety of purified melanin for normal cells as a biocompatible agent as it has shown negligible effect against the normal cell line. On the other hand, the purified melanin pigment was able to reduce the viability of tumor cells in a dose-dependent manner, as shown in Fig. 12. Longer exposure had additional toxicity for the cells. The cell viability of all tested cell lines was remarkably inhibited in the presence of melanin at a concentration of 0.5 μg/mL or higher. The IC_50_ of purified melanin was 108.9, 43.83, and 81.99 μg/mL for HCT116, HEPG, and MCF7, respectively. Importantly, the comparison of all IC_50_ results clearly indicated that the purified melanin demonstrated higher cytotoxicity against cancer cells in comparison with a normal cell line. Therefore, *S. djakartensis* NSS-3 melanin pigment can be used as a potential natural antitumor agent. Similar results were reported on the HEP2 carcinoma cell line by Arun, et al. [120], who reported that inhibition of cell viability was concentration-dependent and that 60 µg of melanin inhibited cell viability by 53%. Without harming or adversely affecting other healthy cells or bodily tissues, melanin offers anti-tumor properties that include apoptosis promotion and angiogenesis inhibition [126]. Melanin has good photothermal conversion efficiency and can absorb light from the ultraviolet to the near-infrared range. This means that it can be used as a photothermal agent in multimodal imaging-guided photothermal therapy (PTT) to target tumor cells or tissues specifically [127].

### Antimicrobial activities

The antibacterial activity of purified melanin was investigated against four MDR bacterial strains, namely *Staphylococcus aureus* (SA-04), *Escherichia coli* (EC-03), *Klebsiella pneumoniae* (KP-01), and *Pseudomonas aeruginosa (*PA-09). The antibacterial activity of the purified melanin in different concentrations (10, 20, 30, 40, and 50 µg/mL) was quantitatively assessed on the basis of zones of growth inhibition. The zone of growth inhibition was accurately measured and compared with the standard antibiotics given in Table 6. Among the Gram-negative bacteria, *P. aeruginosa* was the most inhibited by melanin, with a zone of inhibition of 35.3 ± 0.47 mm at the highest concentration (50 μg/mL) and 11.3 ± 0.0 at the lowest concentration (10 μg/mL). The present study clearly indicates that melanin exhibited strong antibacterial activity against all isolates, even at low concentrations. Melanin showed a considerable amount of microbial growth inhibition, and the values of MIC and MBC are shown in Table 6. MIC values of melanin gave the lowest values of 6.25 µg/mL against *P. aeruginosa* among Gram-negative bacteria and 25 µg/mL against *S. aureus* among Gram-positive bacteria.

**Table 6:** Mean of diameter of inhibition zones obtained by purified melanin pigment against tested bacterial strains

Microbial strains	Mean of diameter of inhibition zone (mm) ± standard deviation									
	Different concentrations of melanin pigment (µg/mL)					MIC (µg/mL)	MBC (µg/mL)	Antibiotic (Positive control)		DMSO (1%) (Negative control)
								Streptomycin (10 µg/mL)	Tetracycline (10 µg/mL)	
	10	20	30	40	50					
Gram-positive bacteria										
*Staphylococcus aureus* (SA-04)	0.0 ± 0.0	9.3 ± 0.47	13.0 ± 0.0	12.0 ± 0.0	19.5 ± 0.02	25	50	30.5 ± 0.05	15.0 ± 0.47	0.0 ± 0.0
Gram-negative bacteria										
*Escherichia coli* )EC-03(	0.0 ± 0.0	0.0 ± 0.0	8.3 ± 0.47	9.6 ± 0.94	12.5 ± 0.05	25	100	25.0 ± 0.00	13.3 ± 0.47	0.0 ± 0.0
*Klebsiella pneumonia* )KP-01(	0.0 ± 0.0	8.3 ± 0.47	11.0 ± 0.0	14.3 ± 0.47	16.0 ± 0.13	12.5	100	27.3 ± 0.47	15.0 ± 0.02	0.0 ± 0.0
*Pseudomonas aeruginosa* )PA-09(	11.3 ± 0.0	14.6 ± 0.94	16.0 ± 0.0	21.0 ± 0.0	35.3 ± 0.47	6.25	25	27.3 ± 0.47	15.0 ± 0.00	0.0 ± 0.0

The melanin pigment showed considerable antimicrobial activity against the tested bacteria, with inhibitory growth zones varying in diameter depending on the bacterial species. As the amount of melanin increased, the diameter of the inhibition zones increased as well. The highest antimicrobial activity was observed against *P. aeruginosa* ATCC 902 *(*PA-09), followed by *S. aureus* (SA-04) and *K. pneumoniae* (KP-01). The least antimicrobial activity was noticed against *E. coli* (EC-03). The MIC and MBC values of melanin on pathogenic bacteria demonstrated that it has the potential to suppress bacterial cell development even at low bacteriostatic concentrations. These findings indicated that biosynthesized melanin had antibacterial activity which might potentially be used in development of novel antimicrobials or antibiotics adjuvants that enhance activity of existing drugs. Antimicrobial assessment results are consistent with those discovered by other authors. Laxmi [128] found that melanin derived from *Providencia rettgeri* hindered the growth of *P. aeruginosa*, and some *Bacillus* species. Xu [129] investigated the antibacterial efficacy of *Lachnum* YM30 melanin and discovered that it was effective against a wide range of microorganisms, including *S. aureus*. According to many studies, the antibacterial properties of melanin may be attributed to its ability to disrupt cell membranes [130]. This disruption can impair the functioning of bacteria, increase the leakage of cell contents, enhance the uptake of non-protein nitrogen, reduce the membrane potential, and inhibit the formation of biofilms regulated by quorum sensing (QS) systems, thereby inhibiting these systems in bacteria [131].

## Conclusions

*S. djakartensis* NSS-3 is a soil-isolated actinobacterium whose melanin pigment has been studied for the first time. In this research, RSM optimization demonstrated the capability of improving this bacterium to produce abundant melanin pigment using L-tyrosine. The phenolic structure of pyomelanin, isolated from *S. djakartensis NSS-3*, was found to have multiple biological properties, including the ability to scavenge free radicals and provide high radioprotection activity. It was found that purified melanin pigment had no cytotoxic effect on healthy cells, demonstrating its safe nature. The results also showed that purified melanin has powerful antimicrobial and antitumor effects against various microorganisms and cancer cell lines. The overall findings of this study showed the potential of pyomelanin as a novel biomaterial with improved biological properties, with further future studies on its mode of action for use in different medical and biotechnological applications.

### Supplementary Information

**Additional file 1: Table S1.** Experimental independent variables at two levels used for the production of melanin by ACT3 using Plackett Burman design. **Table S2.** Experimental variables for Box-Behnken design at different levels. **Table S3.** Cultural characteristics of ACT3 strain on different culture media. **Table S4.** Physiological and biochemical characterization of isolated ACT3 strain. **Table S5.** Physicochemical properties of melanin produced by S. djakartensis NSS-3 and standard synthetic melanin. **Figure S1.** Soil samples collected from Wadi-Allaqui Biosphere Reserve on the eastern side of Lake Nasser, Egypt. **Figure S2.** TLC analysis of purified melanin pigment (**A**) compared with standard synthetic melanin (**B**).

## Data Availability

The datasets generated during and/or analyzed during the current study are available from the corresponding author on reasonable request. All data generated or analyzed during this study are included in this published article (and its Additional file 1).
