# Supplementary material for: Bioproduction and optimization of newly characterized melanin pigment from Streptomyces djakartensis NSS-3 with its anticancer, antimicrobial, and radioprotective properties
Source: Microb Cell Fact. 2024 Jan 17;23:23. doi: 10.1186/s12934-023-02276-y (PMC10792909; doi:10.1186/s12934-023-02276-y)
Supplement: Supplementary file 1 — Additional file 1: Table S1. Experimental independent variables at two levels used for the production of melanin by ACT3 using Plackett Burman design. Table S2. Experimental variables for Box-Behnken design at different levels. Table S3. Cultural characteristics of ACT3 strain on different culture media. Table S4. Physiological and biochemical characterization of isolated ACT3 strain. Table S5. Physicochemical properties of melanin produced by S. djakartensis NSS-3 and standard synthetic melanin. Figure S1. Soil samples collected from Wadi-Allaqui Biosphere Reserve on the eastern side of Lake Nasser, Egypt. Figure S2. TLC analysis of purified melanin pigment (A) compared with standard synthetic melanin (B). [file 12934_2023_2276_MOESM1_ESM.docx]

**Table S1**. Experimental independent variables at two levels used for the production of melanin by ACT3 using Plackett Burman design.

| **Variables** | **Symbol** | **Level** | |
| --- | --- | --- | --- |
|  |  | **Low (−1)** | **High (+ 1)** |
| Incubation period | A | 4 days | 10 days |
| pH | B | 5 | 7 |
| Temperature | C | 30 °C | 40 °C |
| Inoculum size | D | 1 mL/L | 3 mL/L |
| Agitation speed | E | 100 rpm | 200 rpm |
| Yeast extract | F | 0.5 g/L | 1.5 g/L |
| Peptone | G | 3 g/L | 7 g/L |
| Peptic digest | sH | 10 g/L | 20 g/L |
| L-tyrosine | I | 1 g/L | 2.5 g/L |
| Copper sulphate | J | 0.05 g/L | 0.1 g/L |
| Ferric ammonium citrate | K | 0.3 g/L | 0.7 g/L |
| Dipotassium phosphate | L | 0.5 g/L | 1.5 g/L |
| Sodium thiosulphate | M | 0.05 g/L | 0.1 g/L |

Table S2. Experimental variables for Box-Behnken design at different levels

| Factors | Variables | Units | Experimental values | | |
| --- | --- | --- | --- | --- | --- |
|  |  |  | Low level (-1) | Intermediate level (0) | High level (+1) |
| A | L-tyrosine | g/L | 1.00 | 3.25 | 5.50 |
| B | Time | Days | 4.00 | 9.00 | 14.00 |
| C | Ferric ammonium sulphate | g/L | 0.50 | 1.25 | 2.00 |

Table S3. Cultural characteristics of ACT3 strain on different culture media

| Medium | Growth Characteristic | Color of aerial mycelium | Color of substrate mycelium | Color of diffusible pigment |
| --- | --- | --- | --- | --- |
| Peptone yeast extract iron agar (ISP-6) | Heavy | Dusty Gray | Brown | Dark brown |
| Inorganic salts- starch iron (ISP-4) | Heavy | Dark gray | Dark gray | Pale brown |
| Oatmeal (ISP-3) | Moderate | Gray | Brown | None |
| Yeast extract-malt extract (ISP-2) | Moderate | Gray | Beige | None |

Table S4. Physiological and biochemical characterization of isolated ACT3 strain

| Biochemical tests | Results |
| --- | --- |
| Degradation of Starch | + |
| Degradation of Casein | ++ |
| Degradation of Gelatin | + |
| Degradation of Glucose | - |
| Degradation Lactose | - |
| Degradation Saccharose | + |
| Degradation Citrate | +++ |
| H2S production | - |
| Production of Urease | + |
| Mannitol | _ |
| Catalase | ++ |
| Action on Skimmed milk | - |
| Physiological tests | |
| Gram-staining | + |
| Melanoid pigment production on ISP6 | + |
| Growth temperature | 25 +++  35 -  45 -  55 - |
| Optimum pH | 5 +  7 ++++  9 ++  10 + |
| NaCl tolerance (W/V) | 3% +  5% +++  7% +  10% + |

+; Positive, −; negative

Table S5. Physicochemical properties of melanin produced by S. djakartensis NSS-3 and standard synthetic melanin

| Property | Treatment | Results | | |
| --- | --- | --- | --- | --- |
|  |  | Purified melanin | Synthetic melanin | |
| Color | Observation | dark brown | Black | |
| Solubility | Distilled water | Partially soluble | Soluble | |
|  | Methanol | Partially soluble | Soluble | |
|  | Absolute ethanol | Partially soluble | Soluble | |
|  | Chloroform | Partially soluble | Soluble | |
|  | Benzene | Partially soluble | Soluble | |
|  | Acetone | Partially soluble | Soluble | |
|  | Ethyl acetate | Partially soluble | Soluble | |
|  | 1Mol/L KOH | Soluble | Soluble | |
|  | 1Mol/L NaOH | Soluble | Soluble | |
|  | DMSO | Soluble | Soluble | |
| Stability | Heat (60° C for 2, 4, 6 h, respectively) | Stable | Stable | |
|  | Heat (80° C for 2, 4, 6 h, respectively) | Stable | Stable | |
|  | Heat (100° C for 2, 4, 6 h, respectively) | Stable | Stable | |
|  | Sun light (treatment for 10 days) | Stable | Stable | |
| Precipitation with | 3N HCl | Precipitated | | Precipitated |
|  | 1% FeCl3 | Precipitated | | Precipitated |
| Oxidation | Hydrogen peroxide | Decolorization (oxidized) | | Decolorization (oxidized) |
| Bleach test | KMNO4 | Decolorization | | Decolorization |


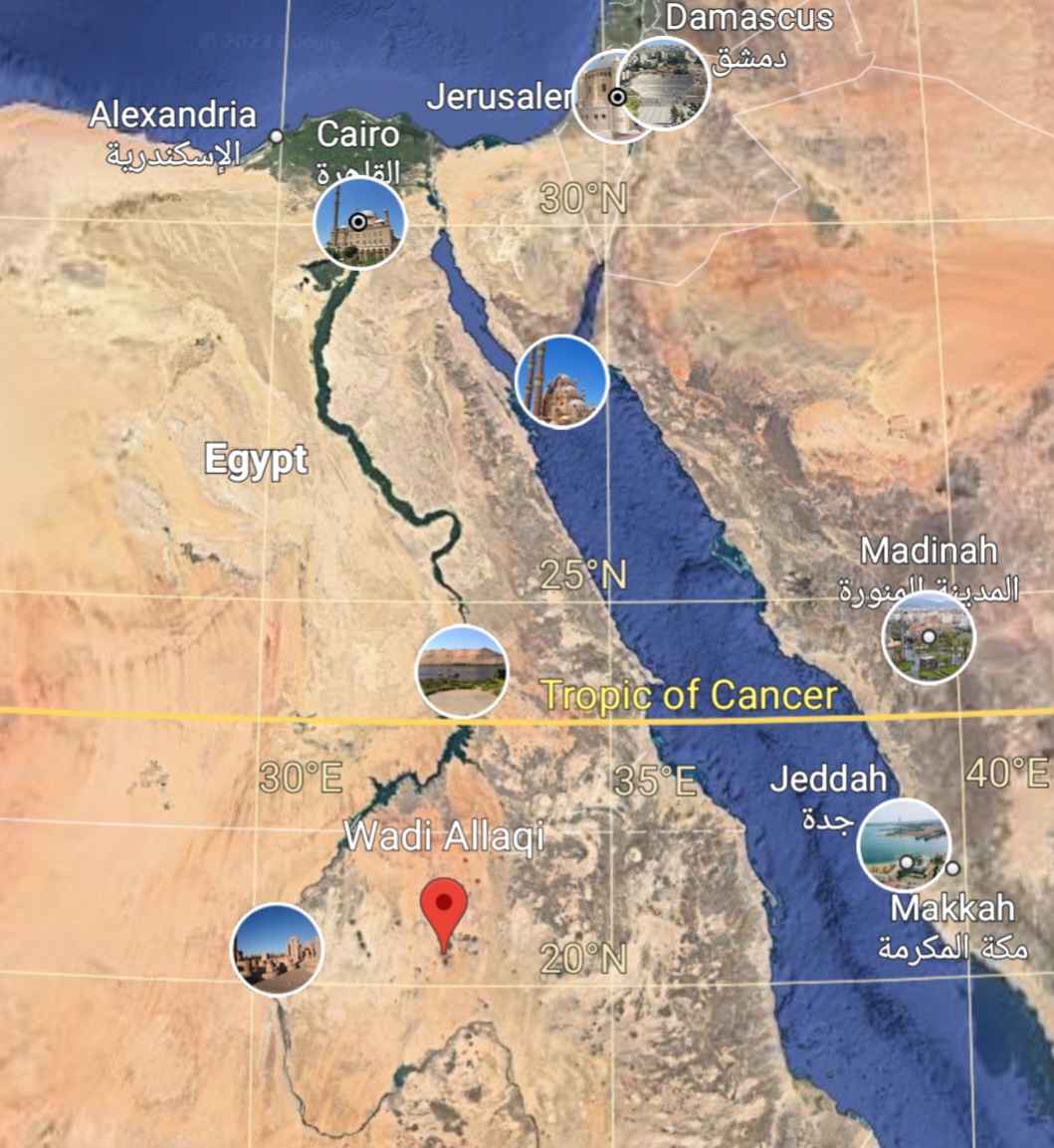


Fig.S1 Soil samples collected from Wadi-Allaqui Biosphere Reserve on the eastern side of Lake Nasser, Egypt.


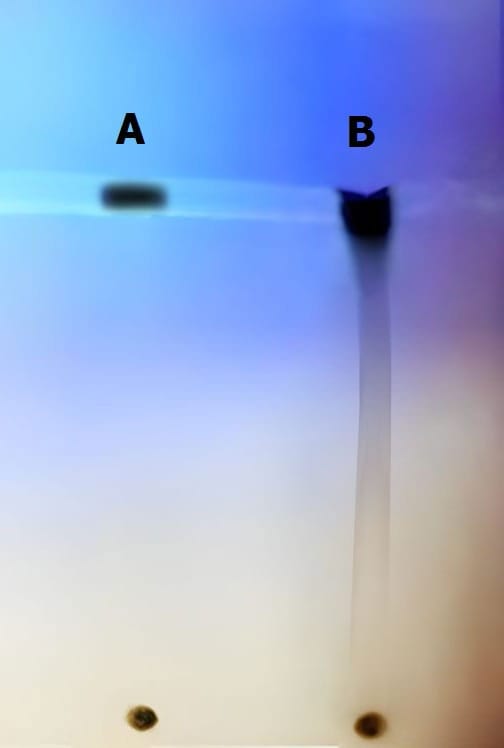


Fig.S2 TLC analysis of purified melanin pigment (A) compared with standard synthetic melanin (B).
